# Real-time genome imaging of host interactions in adeno-associated virus genome release

**DOI:** 10.1016/j.isci.2025.112624

**Published:** 2025-05-08

**Authors:** Luisa F. Bustamante-Jaramillo, Lei Yue, Joshua Fingal, Gustaf Rydell, Maria Johansson, Tomas Edreira, Oliver J. Müller, Susanne Hille, Martin Müller, Franck Gallardo, Qingxin Chen, Marie-Lise Blondot, Michael Kann

**Affiliations:** 1 Department of Infectious Diseases, Institute of Biomedicine, Gothenburg University, Gothenburg, Sweden; 2 Department of Internal Medicine V, University of Kiel, and German Centre for Cardiovascular Research, Partner Site Hamburg/Kiel/Lübeck, Germany, Kiel, Germany; 3 Infection, Inflammation and Cancer Program, German Cancer Research Center (DKFZ), Heidelberg, Germany; 4 Neo Vir Tech SAS, Centre Pierre Potier, Toulouse, France; 5 CNRS UMR 5234, Université de Bordeaux, Bordeaux, France; 6 Sahlgrenska Academy, Gothenburg, Sweden; 7 Department of Chemistry and Molecular Biology, Gothenburg University, Gothenburg, Sweden

**Keywords:** Cellular therapy, Genomics, Virology, Genomic analysis

## Abstract

Adeno-associated virus (AAVs) with a self-complementary genome (sc) comprising a gene of interest are used in gene therapy. Their efficiency is limited but the molecular factors contributing to this restriction are poorly understood. We utilized scAAV2 containing a fluorescent protein-binding anchor sequence on its genome allowing visualization of released genomes by time-lapse microscopy. Pairing this technique with capsid staining, we showed that scAAV2 genome release was initiated by a partial genome exposure, triggered by elevated calcium levels while the capsids interacted with proteins of the nuclear pore. Genome release occurred subsequently requiring Rad52 decamerization in the vicinity of the host chromatin. A fraction of the released genomes was degraded by Mre11, an essential factor for chromatin stability, and cellular DNA double-strand breaks. These steps were key-factors limiting transduction, suggesting that temporary modulation of DNA damage-response-proteins is a promising way to increase scAAV efficiency in therapy.

## Highlights

• Real-time imaging of scAAV2 genome release in living cells
• Elevated Ca^++^ primes AAV2 for genome release while interacting with the NPC
• Rad52 decamerization promotes genome release near the host chromatin
• Released AAV2 genomes are degraded by Mre11

## Graphical abstract

**Figure:**
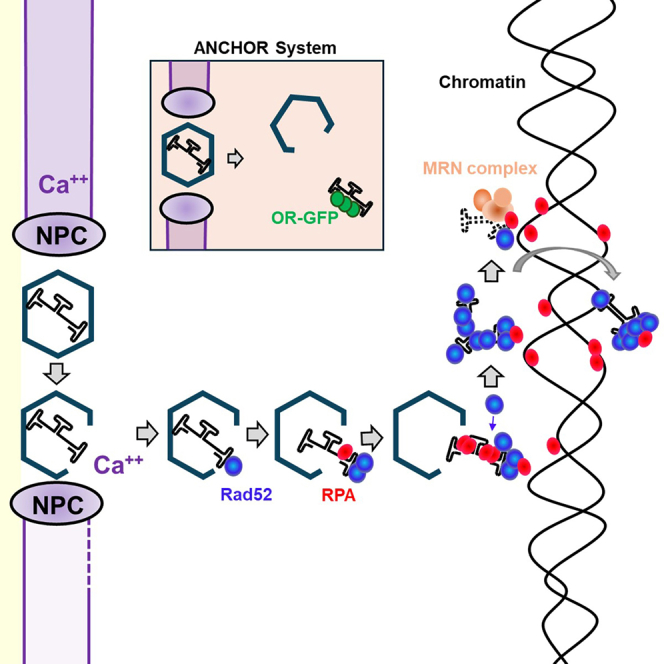

## Introduction

Uncoating of viruses is intricately linked to the nature of their genome, which is intertwined with their place of replication. While practically all RNA viruses replicate within the cytoplasm^1^ (with exceptions like influenza and bornaviruses), DNA- and retroviruses replicate within the nucleus^2^ (with poxviruses being a notable exception^3^). In consequence, DNA viruses have to transport their genome toward the nuclear envelope (NE) within a protein shell, often forming a (nucleo)capsid.^2^

Most virus capsids exceed the 39 nm functional diameter of the nuclear pore complex (NPC)^4^ and disassemble at its cytoplasmic face.^5^ For example, herpes virus capsids (120 nm) bind to the NPC via receptors of the importin β family,^6^^,^^7^ opening at the opposite site to inject their genomes while remaining intact. In contrast, adenovirus capsids (90 nm) also bind to the NPC but disintegrate into smaller entities,^8^ allowing the genome complex with adenoviral proteins to be transported through the NPC via nuclear transport receptors.^2^

A few viruses exhibit capsids with diameters smaller than 39 nm. Among them are parvo- and hepatitis B viruses with diameters of 20–25 nm and 36 nm, respectively. Hepatitis B virus capsids pass through the nuclear pore mediated by importins, followed by tight binding to the nuclear part of the NPC where they disintegrate.^9^ In contrast, the fate of parvoviruses remains largely unsolved.

This knowledge gap is particularly striking given the medical relevance of parvoviruses, such as their use as oncolytic agents—exemplified by H1-PV in glioblastoma treatment (ParOryxV)^10^ and their role as vectors in gene therapy; the latter primarily utilizing adeno-associated viruses (AAVs) with a modified self-complementary genome (scAAV). Through 2023, scAAVs have been employed in 136 clinical trials^11^ and approved for six therapies by the FDA and EMA for the treatment of various monogenic disorders. However, these vectors still exhibit limited transduction efficiency, requiring doses of 10^12^–10^13^ scAAV/kg of body weight. This highlights the necessity of understanding the rate-limiting steps in the intracellular processing of scAAV to enhance therapeutic outcomes.

The capsids/coats of scAAVs are structurally identical to wild-type AAVs (wtAAVs), consisting of three structural capsid proteins (VP1, VP2, and VP3) and rep78 – termed NS1 in other parvoviruses. Rep78, which is absent in scAAVs, is covalently bound to the 5′ end of the genome and is exposed on the exterior of the virion.^12^^,^^13^^,^^14^^,^^15^^,^^16^ The AAV wt genome is a linear DNA of ∼4–6 kb,^16^^,^^17^^,^^18^^,^^19^^,^^20^ with palindromic ends forming hairpins (inverted terminal repeat, ITR). They contain the origin of replication and the genome packaging signal,^21^ and they are required for genome circularization.^22^ Replication of wtAAVs relies on co-infection with a helper virus, e.g., adeno- or herpes simplex viruses, which are required for genome replication and virus propagation but not for entry into the target cell.^23^^,^^24^

**Figure 1:**
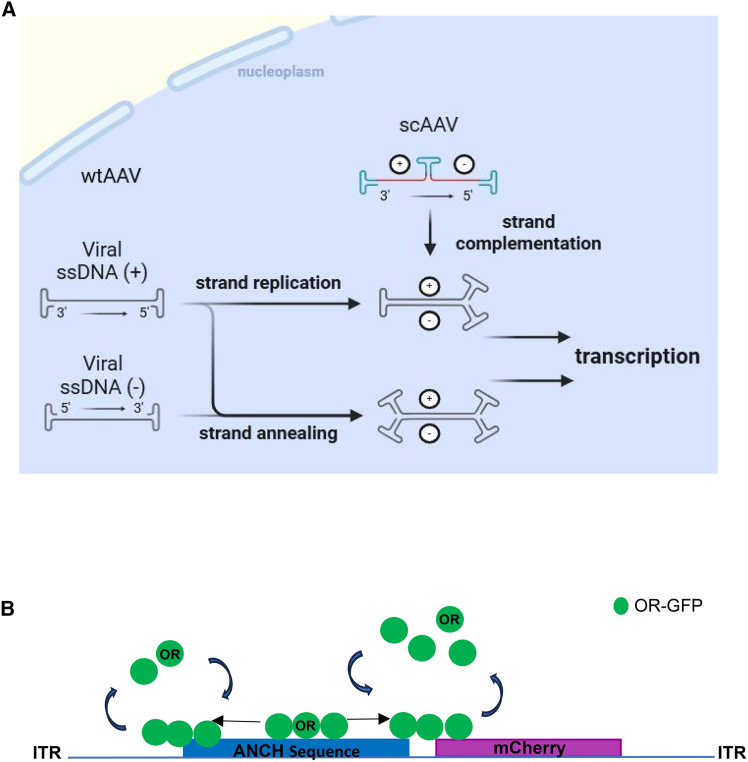
Scheme of AAV genome structure (A) AAV genome processing required for transcription. Left: wtAAV, right: scAAV (modified from McCarty et al., 2008). Blue: ITRs, Red: gene of interest. (B) Graphical description of scAAV2-ANCH-mCherry system. The OR-GFP protein expressed in U2OS cells binds to the dsANCH sequence upon genome release.

The replacement of the wt single-stranded DNA by artificial double-stranded genomes allows the by-passing of genome resolution during replication and co-infection–restricted expression. Within the virion, these genomes exhibit a single-stranded linear conformation^25^ (Figure 1A).

AAVs – as other parvoviruses – enter the cell by clathrin-mediated endocytosis.^26^^,^^27^ Endosomal acidification exposes a phospholipase A_2_ (PLA_2_) domain and a nuclear localization sequence (NLS). PLA_2_ activity is crucial for capsid escape from endocytic vesicles,^28^^,^^29^^,^^30^^,^^31^ while the NLS facilitates the recruitment of nuclear transport receptors of the importin family during active capsid translocation to the nucleus.^32^ The importance of importin binding to the capsids can be concluded from the fact that microinjection of wheat germ agglutinin (WGA) blocks infection with CPV.^33^ WGA prevents the binding of importin β to proteins of the NPC, thus inhibiting nuclear import of various cargos.^34^ Both endosomal escape and nuclear entry were described to be rate limiting in the infection process.^35^^,^^36^^,^^37^^,^^38^ In agreement with these observations, nuclear capsids from different parvoviruses were visualized within 2 h post infection,^26^^,^^39^^,^^40^^,^^41^ requiring importin β (CPV^42^), although alternative nuclear transport receptors were described (AAV^43^). However, the nuclear genome arrival could not be attributed to the presence of nuclear capsids, as genome liberation could have occurred prior to nuclear capsid arrival.

The direct connotation of a nuclear import of genome-containing intact capsids through the NPC was questioned by observations using H-1PV, CPV, and AAV2, showing that capsids induce transient pores in the NE,^44^^,^^45^^,^^46^^,^^47^ through which the capsids may pass through passive diffusion. Pore formation was shown to also depend on capsid interaction with the NPC,^47^ but required mitotic enzymes such as protein kinase C α and cyclin-dependent protein kinases. Their activation was found to depend on elevated cytoplasmic Ca^++^ concentrations, presumably released from PLA_2_-mediated holes in the NE.^47^

A few studies investigated parvoviral genome release from the capsids.^48^^,^^49^ Indirect data using recombinant AAV with single stranded genome (rAAV) showed that double-strand formation occurred 3 h–8 h post infection, followed by progressive genome disappearance.^48^

Further, the appearance of small nuclear CPV capsid entities at some distance from NE^32^ suggests nuclear genome release by capsid degradation. However, *in vitro* experiments using heat treated wtAAVs indicated that a linear ejection of genomes from nearly intact capsids may also occur.^50^ Consequently, host factors driving genome release remain unknown, requiring either interactions with the capsids for degradation, or interacting with the viral genome after initial capsid opening. Due to the hairpin structure of the genome ends, the latter scenario could potentially include DNA damage response (DDR) proteins, including the Mre11-Rad50-Nbs1 (MRN) complex, which is needed for efficient scAAV genome circularisation.^22^ In non-infected cells, the MRN complex is directly involved in DNA double-strand break repair by coordinating the break ends and initiating end-resection.^51^

Amongst the DDR proteins is also the radiation sensitive 52 (Rad52), which binds directly or indirectly to AAV ITRs and promotes AAV transduction.^48^^,^^52^^,^^53^ Physiologically, Rad52 binds predominantly to single-stranded DNA forming a homodecamer^54^^,^^55^^,^^56^^,^^57^ upon interaction with replication protein A complex (RPA),^58^ which comprises RPA1 (also termed RPA70), RPA32 and RPA3.^59^^,^^60^ Of note, the crucial Rad52 decamerization can be targeted by different inhibitors addressing distinct functions of the complex, also avoiding misinterpretation of off-target effects. D-I03 binds to the N-terminal domain of Rad52,^61^ which is required for forming the homododecamer.^62^ HAMNO interacts with the N-terminal domain of RPA70 and impairs RPA32^63^ by inhibiting its phosphorylation. This in turn is required for interaction of the RPA complex with RAD52,^64^^,^^65^ without affecting binding of the complex to DNA.^63^

In order to unravel the molecular mechanisms of AAV genome release from the capsid, which are inextricably linked to preceding events at the NE, we investigated the kinetics of genome release by inhibiting potential key enzymes needed for AAV nuclear import and genome release.

We applied scAAV2, in which the AAV ORFs are replaced by a fluorescent protein expression cassette and an anchor sequence (ANCHOR; scAAV2-ANCH-mCherry) (Figure 1B). This allows us to monitor successful transduction and to detect released genomes by time-lapse microscopy. The ANCHOR system^66^ consists of the heterologously expressed bacterial OR protein fused to EGFP, which binds to the dsDNA anchor sequence (ANCH). The binding spreads to neighboring sequences by EGFP-OR multimerization to >400 copies. EGFP-OR binding to ANCH is dynamic with a half-life of 52 s,^67^ indicating a rapid recycling of EGFP-OR protein onto the tagged viral DNA. This approach allowed us to show intercellular variations of scAAV2 infections upon the application of different inhibitors, targeting Ca^++^ storage or the activity of different DDR proteins. The results made it possible to identify novel factors involved in scAAV2 uncoating and led to the proposal of a unique strategy of viral genome release.

## Results

### Kinetics of scAAV2 genome liberation and mCherry expression

**Figure 2:**
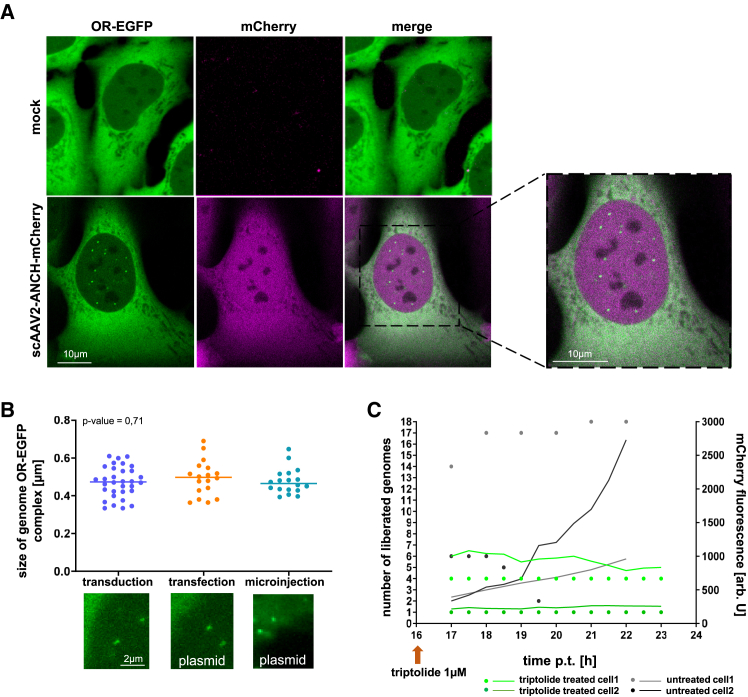
Detection of ANCH sequences in OR-EGFP expressing cells (A) Confocal image of U2OS-OR-EGFP (green) non-transduced and transduced cells with AAV2-ANCH-mCherry. Green: OR-EGFP, magenta: mCherry. The genomes appear as intranuclear foci. Right panel: magnification of the indicated cell. Scale bar 10 μm. (B) Upper panel: foci size in transduced, transfected and microinjected cells. Y axis: size in μm. The *p*-value on top of the panel indicates the no significant difference (Kruskal-Wallis test). The bars are the median. Lower panels: Examples of the foci. Scale bar 2 μm. (C) Time course of scAAV2 genome release and mCherry expression in untreated (gray) and triptolide-treated cells (green) recorded by LSM. Left y axis: number of released genomes. Each dot represents the number of released genomes per cell. Right y axis: mCherry expression in arbitrary units. Triptolide was added at 16 h p.t., as indicated by the arrow. For quantification in higher cell numbers at 16 h p.t. see Figure S2C. The figure shows the quantification in different cells in 2 independent experiments.

Transduction of OR-EGFP U2OS cells with 100 viral genome equivalents (vg)/cell scAAV2-ANCH-mCherry resulted in distinct foci as early as 2 h post transduction (p.t.) (Figure 2A, Video S1; Figure S1A), exhibiting variable diameters of 0.47 μm on average by laser scanning microscopy (LSM) (Figure 2B). No distinct size classes were observed, suggesting the absence of defined clusters containing one or more genomes. Furthermore, no statistically significant difference in size was observed after transfection or microinjection of the plasmid containing the scAAV2-ANCH-mCherry genome (Figure 2B). Transduction-induced foci were strictly intra-nuclear (Figures 2A and S1A), consistent with intra-nuclear release of AAV genomes. No foci were visible in MOCK-infected cells, and mCherry expression was strictly associated with the appearance of foci. mCherry expression could be observed at 8 h–12 h post-transduction and continued throughout the observation periods in foci-positive cells (Figure S2A). Consistent with the turnover of OR-EGFP, no bleaching of the foci was observed (Figure S2B). In contrast, other non-transduction-related fluorescent clusters, such as those from OR-EGFP aggregates, were bleached.

Video S1. OR-EGFP-U20S cells transduced with scAAV2-ANCH-mCherry 30 h, 1 h per frame. Projection based on nine 8 μm z stacks adjusted to the central cell. Released genomes are marked with triangles. Green: OR-EGFP, magenta: mCherry. The central cell follows a class III kinetic with disappearing genomes.

Inhibition of transcription initiation (RNAP I and RNAPII) with triptolide, after established mCherry expression at 16 h p.t., resulted in a block of mCherry expression (Figure 2C). This blockage did not affect the appearance of additional genome foci (Figures 2C and S2C). We thus excluded a transcription-caused displacement of OR-EGFP from the genome that could prevent genome detection.

Genome signals were nuclear OR-EGFP expression dependent, but independent of time p.t. (Figure S2D). After microinjection of scAAV2-ANCH-mCherry genome-containing plasmid into the cytoplasm of OR-EGFP U2OS cells, and within 30 s, we could observe the onset and maximum signal strength. This indicates that viral genomes became visible immediately after release (Figure S2E).

**Figure 3:**
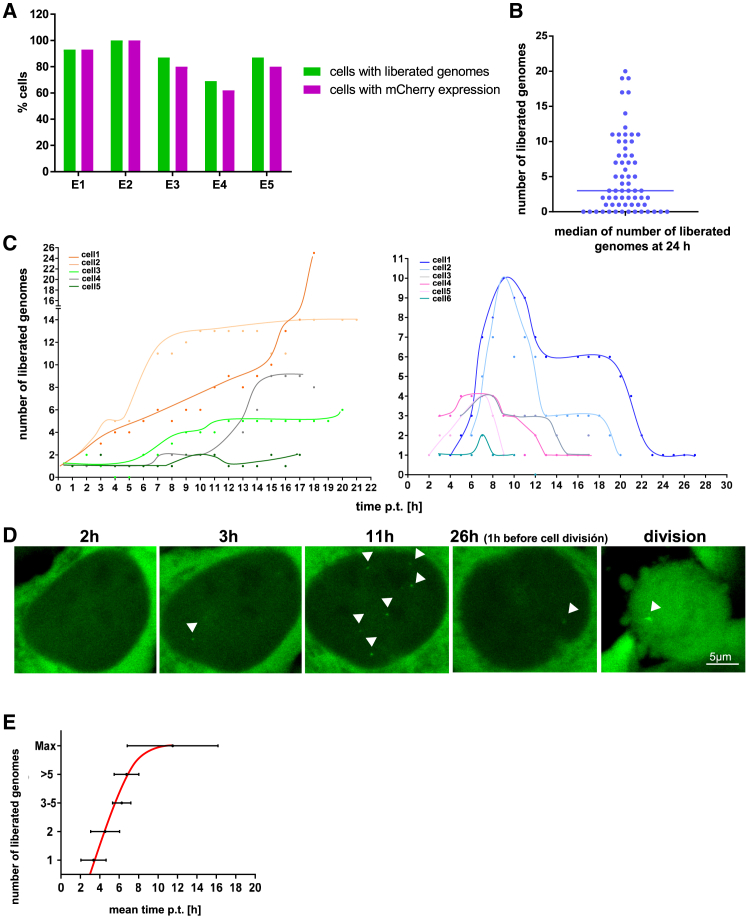
Kinetic of liberated scAAV2 genomes (A) Transduction efficiency. Y axis: percentage of mCherry-expressing cells, which exhibited released genomes at 20 h p.t. (5 independent experiments [E1 – 5] using different capsid preparations). (B) Number of liberated genomes per cell at 24 h p.t. X axis: time p.t., Y axis: number of released genomes per cell. The median is indicated as a horizontal bar. The graph is based on data from 65 cells from 4 experiments. (C) Kinetics of AAV2-ANCH-mCherry uptake and release, exemplified in 10 cells from 5 experiments. Left: continuous uptake (class II), right: disappearance after plateau (class III). The released genomes in each cell are indicated in different colors. Each dot indicates the number of foci. X axis: time after transduction, y axis number of liberated genomes. (D) LSM of an example class III cell following a decline of released genomes. The foci indicating released scAAV2-ANCH-mCherry genomes are labeled with white triangles. Scale bar 5 μm. (E) Mean time of first appearance and the maximum number of released genomes. The error-bars indicate standard-deviation from different cells (5 cells from 5 experiments).

At transduction with 100 vg/U2OS cell we observed that 70–100% of cells showed released genomes, while mCherry expression was found in 89–100% of cells with released genomes (Figure 3A). The number of liberated genomes at 24 h was in a range of 1–20 genomes per cell, with a median of 3 copies (Figure 3B).

For evaluation of genome liberation efficiency, we first controlled the intracellular scAAV2-ANCH-mCherry genome distribution by subcellular fractionation following digital droplet PCR (ddPCR) at 24 h p.t. We observed that 40% genomes/genome-containing capsids (average 40 genomes per cell) bound or entered the cells (Table 1). 48% of these (19 genomes per cell) entered the nucleus or were associated with the NPC. Three genomes were found to be chromatin-associated (16%), which is identical to the number of released viral genomes observed by real-time microscopy (Figure 3B). Collectively, these quantifications suggest that, although capsid trafficking to and into the nucleus was restricted, the rate-limiting step seems to be intranuclear genome liberation, at least throughout the 1-day observation period.

**Table 1:** Intracellular scAAV2 genome distribution by digital droplet PCR after cellular fractionation

scAAV2-YFP		vg		vg/cell	
		ddPCR1	ddPCR2	ddPCR1	ddPCR2
cell	CE	1,9E+06	2,1E+06	2	2
	ME	1,8E+07	1,8E+07	18	18
	NP	1,3E+07	1,9E+07	13	19
	CB	3,0E+06	2,8E+06	3	3
	PE	5,2E+05	6,3E+05	1	1
	Total	3,7E+07	4,2E+07	37	42
nuclei	(NP+CB)	1,6E+07	2,2E+07	16	22

Cells were transduced with 100 scAAV2-YFP/cell for 2 h and harvested 24 h p.t. CE: cytosol, ME: membranes, NP: nucleoplasm including nuclear pores, CB: chromatin-bound proteins, PE: cytoskeleton. The fractionation control is given in Figure S4H.

By following the fate of the released genomes with time-lapse microscopy we distinguished three classes of cells. Class I cells were refractory to transduction (7/64 cells, 11%), although some experiments yielded higher or lower numbers (Figure 3A). Class II cells exhibited accumulation or stable number of released genomes for 20 h during which no cell division occurred (15/64 cells, 23%). In class III cells visible genomes disappeared prior to cell division (42/64 cells, 66%) (Figures 3C and 3D).

In class II and III cells, genome foci became visible from 2 h p.t. onwards, with a mean appearance time of 3.5 h, reaching a maximum after 16 h (Figure 3E). While visible, the intensity of the genome foci showed only small variations (Figure S2I). mCherry expression was also variable, and we could not observe any correlation between the amount of mCherry expression and the number of released genomes at both 8 h and 14 h p.t. (Figure S2F).

In class III cells, the disappearance of the genome signal started at 6 to 11 h p.t. (Figure 3C). The half-life of silencing or disappearance was on average 9 h after the peak number of released genomes was observed. Notably, this occurred as early as 5 h before cell division, suggesting that this phenomenon is not primarily driven by cell cycle progression.

To distinguish whether the genomes were degraded or became undetectable, we isolated the nuclei of U2OS cells transduced with scAAV2-Enhanced Yellow Fluorescent protein (EYFP) for 1 h, 8 h, and 24 h. Quantification of nuclear genomes by droplet digital PCR revealed that the total number of viral genomes were 2.8-fold higher at 8 h than 24 h (Figure S2G), suggesting that two-thirds of the liberated genomes were indeed lost by intracellular degradation.

### scAAV2 genomes are released within the nucleus

**Figure 4:**
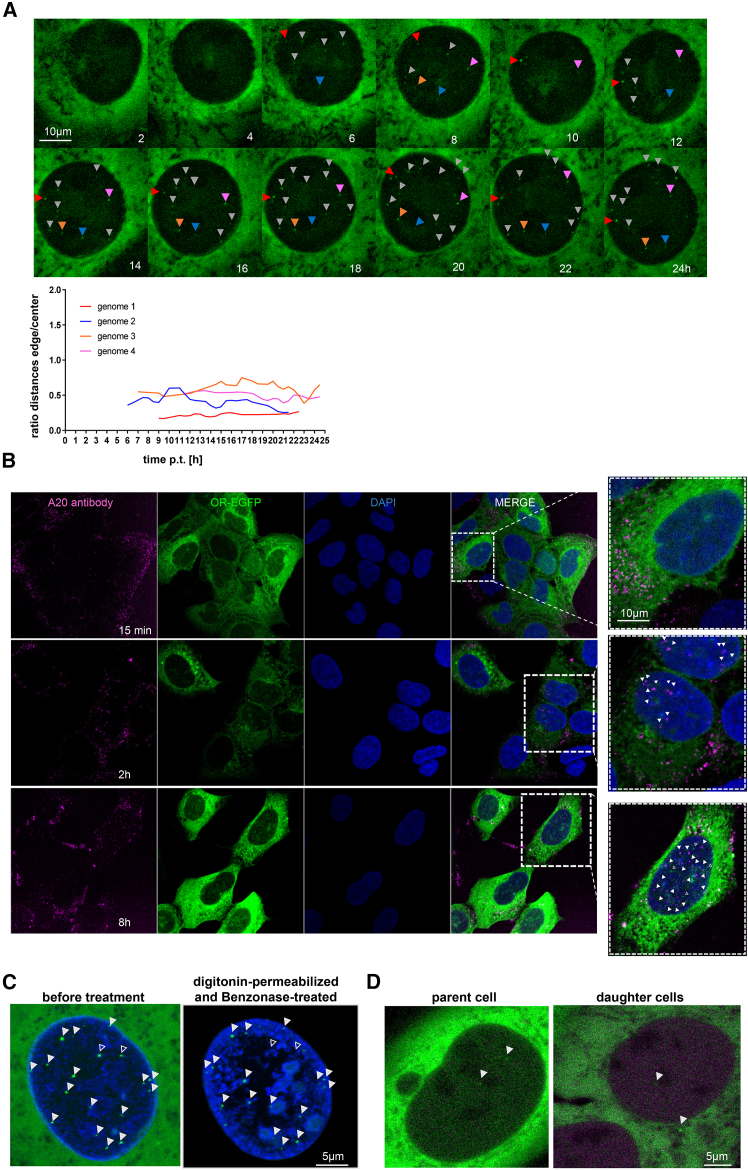
Immobility of scAAV2 genomes and association with chromatin (A) Upper panels: Time-lapse LSM showing released scAAV2-ANCH-mCherry genomes, indicated by white triangles. Maximum projection from 9 z-stacks: of total 8 μm. The time p.t. is indicated on the individual panel [h]. Lower panel: x axis: time p.t. [h]; y axis: quantification of the ratio of the distances of the genomes to the edge of the nucleus and to the nuclear center. The colors of the lines correspond to the genomes indicated by triangles in the upper panel. Scale bar 10 μm. (B) Immunostain of scAAV2-ANCH-mcherry capsids by A20 antibody at 15 min, 2 h and 8 h p.t. scAAV2 was removed at 2 h p.t. by washing. Capsids: magenta, OR-GFP: green, chromatin: blue. The capsids in the magnification on the right panels are indicated by white triangles. Scale bar 10 μm. (C) Localization of scAAV2-ANCH-mCherry genomes adjacent to chromatin after digitonin permeabilization and low-concentration benzonase-treatment for 20 min. Filled arrows: genomes present before and after treatment, empty triangles: genomes sensitive to benzonase. Green: OR-EGFP; blue: Hoechst (see also Video S4). Scale bar 5 μm. (D) Localization of scAAV2 genomes after cell division. Left: cell before division (parent cell), right: after division (daughter cell). The genomes are indicated by white arrows. Scale bar 5 μm.

Liberated genomes appeared to be stochastically distributed throughout the entire nucleus (Figure 4A, upper panel). No accumulation was observed in or close to nucleoli, where numerous proteins from various viruses localize^68^ and where AAV2 capsid assembly occurs.^69^ Furthermore, no accumulation of foci was observed at the NE, suggesting that scAAV2 does not disassemble at the NPC but deeper within the nucleus. These conclusions were supported by a kinetic of intact capsid-visualization by immune staining. We applied the antibody A20, which is established to bind to intact capsids but not capsid subunits.^70^ At 15 min p.t. all intact capsids were found in the cytoplasmic periphery (Figure 4B, upper panel). At 2 h p.t. 12 capsids on average were found within the nucleus, which increased to 20 at 8 h p.t. (Figure 4B, middle and lower panel; Figure S2H), although some capsids remained at the NE. On average, one released nuclear genome was observed at 2 h p.t. (Figure 3D).

Signal intensity of the released genomes did not increase over time (Figures S2B, S2G, and S2I). We thus further conclude that genome liberation, which allow OR-EGFP to accumulate, takes less than 10 min, since this was the shortest interval between frames in our experiments (Figure S2J). Notably, the adjustment of genome fluorescence by the intra-nuclear OR-EGFP fluorescence resulted in mostly identical genome fluorescence (Figure S2J). In contrast, the size of the genome-derived foci varied during time-lapse (Figure S2K), indicating different genome stages.

### scAAV2 genomes bind to nuclear structures

Following the spatial distribution of the genomes over time, we observed that all released genomes exhibited poor mobility relative to the NE (Figure 4A, lower panel), and that genome displacements were rather caused by movement of the entire cell (Video S2). This assumption is consistent with their binding to chromatin previously shown by cellular fractionation, and was further supported by the constant distance to promyelocytic leukemia protein (PML) nuclear bodies (Figures S3A and S3B), without showing co- or nearby localization. Further support for an association with chromatin was obtained by direct visualization of the released genomes and chromatin. After transduction for 24 h, we permeabilized cellular plasma membranes by digitonin, which leaves the nuclear membrane intact although soluble nuclear proteins diffuse out of the nucleus.^71^ This step was followed by Benzonase treatment at low concentrations in order to reduce the nuclear viscosity.^72^ Figure 4C and the Videos S3, and S4 show a co-localization of the released scAAV2 genomes with chromatin, arguing against a passive immobilization due to its size. Since transfected plasmids containing the genome showed the same spatial arrest (Figure S3C), we further conclude that the genome and not potentially attached capsid proteins interact with nuclear structures.

Video S2. OR-GFP-U20S cells transduced with scAAV2-ANCH-mCherry Time course, 30 frames, 30 min per frame, z-stacks: 9 slides (8μm). Image from one slide. The nucleolus is drawn to follow the movement of the cells, and two genomes are followed by the line, blue and yellow.

Video S3. Maximal projection digitonin-permeabilized U2OS-OR-EGFP cells transduced with scAAV2-ANCH-mCherry during chromatin-digestion with Benzonase Z stacks: 5 slides, total 4 μm. Blue: condensed chromatin stained by Hoechst.

Video S4. 3D reconstruction of scAAV2-ANCH-mCherry transduced and digitonin permeabilized U2OS-OR-EGFP cells after 20 min chromatin digestion with Benzonase Z-stacks: 32 slides, total 9 μm. Blue: Hoechst stain.

### scAAV2 genomes dissociate from nuclear structures upon cell division

During the time-lapse experiments, we observed 12 scAAV2-ANCH-mCherry genome-containing cells dividing 18–24 h after removal of the transducing virus. As shown in Figure 4D, cell division resulted in the presence of both nuclear and cytoplasmic genomes in the daughter cells. This finding indicates that the genomes were not actively encapsidated into the nucleus of the daughter cells, but rather passively trapped upon formation of the nuclei during mitosis. This hypothesis argues against a fixation to chromatin during cell division, which would have led to nuclear localization in the daughter cells. This hypothesis was supported by the dissociation of scAAV2 genomes and chromatin in mitosis as shown in Videos S5 and S6 (Video S7: non-transduced cell). mCherry expression was observed in daughter cells with nuclear genomes, suggesting that the genomes were not silenced during cell division (Table S1). Since the expression of mCherry in daughter cells showed a correlation with the expression in the parent cells, it is possible that the lack of correlation between mCherry expression and the number of genomes released may be influenced by genetic factors that vary between individual cells, but which are passed on to daughter cells.

Video S5. scAAV2-ANCH-mCherry-transduced U2OS-OR-EGFP cells during division at 24 h p.t. (cell1) Green: OR-EGFP and genomes Magenta: Rad52 immune stain. Blue: DAPI. The movies show 9 z-stacks covering 8 μm.

Video S6. scAAV2-ANCH-mCherry-transduced U2OS-OR-EGFP cells during division at 24 h p.t. (cell2) Green: OR-EGFP and genomes Magenta: Rad52 immune stain. Blue: DAPI. The movies show 9 z-stacks covering 8 μm.

Video S7. U2OS-OR-GFP non-transduced cells during division at 24 h p.t. (control of Videos S5 and S6)

### Ca^++^ initiates scAAV2 genome release

To distinguish between direct passage of the unmodified capsid through the NPC and the requirement for Ca^++^, U2OS cells were treated with thapsigargin and transduced with scAAV2-EYFP. Thapsigargin prevents Ca^++^ accumulation in the ER,^73^^,^^74^ which is continuous with the lumen between the inner and outer nuclear membranes. As the ER-Ca^++^ concentration is cell cycle–dependent,^75^^,^^76^^,^^77^ we further investigated HUVEC cells, which are primary cells that do not undergo permanent cell division^78^ as U2OS cells do.

**Figure 5:**
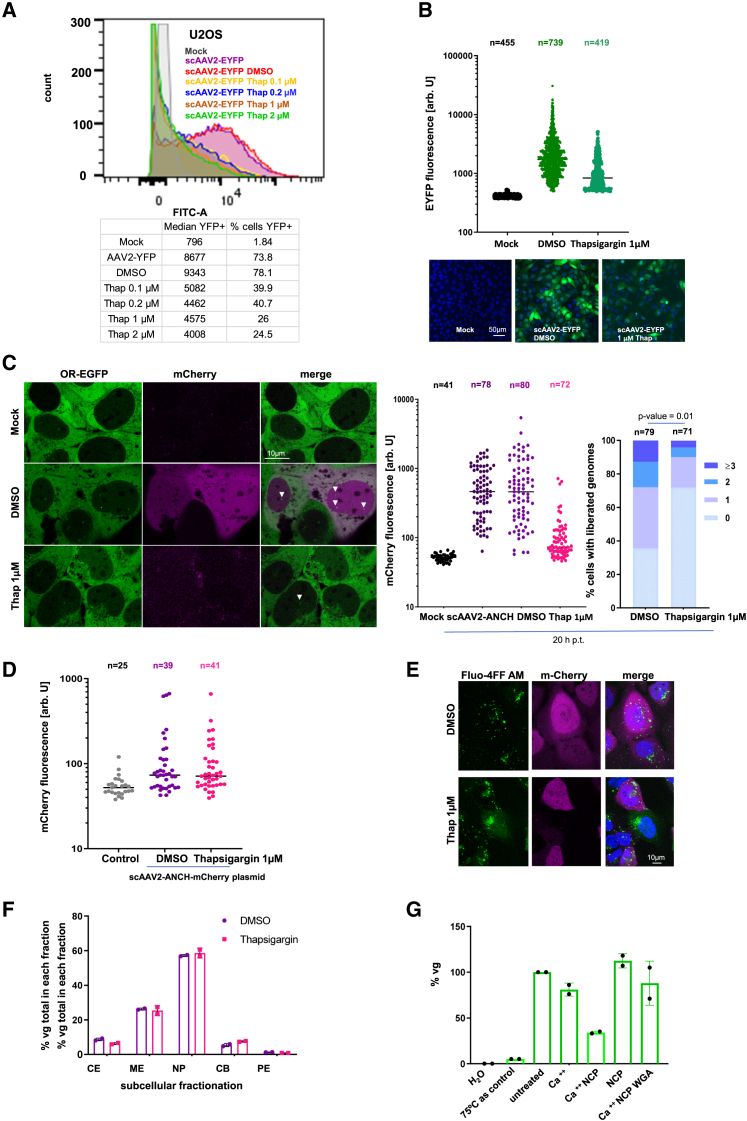
Ca^++^ promotes scAAV2 nuclear genome release (A) FACS analysis of cells pre-treated with thapsigargin for 2 h and transduced with scAAV2-EYFP for 1 h. The analysis was done 24 h p.t. Upper panel: FACS analysis. X axis: EYFP intensity, y axis: cell count. The conditions are given in the panel. Lower panel: quantification of EYFP expression and EYFP positive cells. (B) Upper panel: quantification of EYFP expression from LSM images 24 h p.t. Independent values showing the median and distribution of the signal. n: number of analyzed cells from 6 images. The conditions are indicated on the x axis. Lower panel: LSM image. Scale bar 50 μm. (C) Left panels. Images of OR-EGFP-U2OS cells treated with 1 μM thapsigargin and DMSO, transduced with scAAV2-ANCH-mCherry for 24 h. Green: OR-EGFP, magenta: mCherry. The arrows indicate examples of released genomes. Middle panel: Independent values showing the quantification of mCherry intensity (y axis, arb. U). The conditions are indicated on the x axis. n gives the number of analyzed cells from 10 images. The bar is the median. Scale bar 10 μm. Right panel: quantification of released scAAV2-ANCH-mCherry genomes in % of transduced cells. The colors correspond to the number of released genomes, indicated on the right of the panel. (D) mCherry expression in thapsigargin-treated cells transfected with scAAV2-ANCH-mCherry plasmid for 24 h. Y axis: mCherry fluorescence in arb. U. n: number of analyzed cells from 8 images. (E) LSM images depicting the free intracellular Ca^++^ distribution using Fluo-4FF, AM in U20S cells pre-treated for 1 h with thapsigargin and transduced for 1 h with scAAV2-ANCH-mCherry (confocal microscopy). Green: Ca^++^, magenta: mCherry. Scale bar 10 μm. (F) Quantification of scAAV2-EYFP genomes in different compartments after subcellular fractionation 24 h p.t. by digital droplet PCT. Y axis: % of intracellular genomes. CE: cytosolic, ME: membranes, endosomes, NP: nucleoplasm including nuclear pore proteins, CB: chromatin-bound proteins, PE: cytoskeleton. The purification controls are depicted in Figure S4H. The error-bars indicate standard-deviation from independent duplicates. (G) *In vitro* assay of genome accessibility by DNase I. Y axis: percentage of nuclease-resistant scAAV2-EYFP genomes quantified by droplet PCR. 75C: control using 75°C-pre-treated capsids for liberating the genomes, WGA: wheat germ agglutinin. The error-bars indicate standard-deviation from independent duplicates.

The cells were pre-treated with thapsigargin at non-cytotoxic concentrations for 2 h (Figure S4A). This was followed by transduction with scAAV2-EYFP for 1 h in presence of the inhibitor, which was kept for 20 h. Flow cytometry analysis (FACS) showed a dose-dependent reduction of the number of EYFP-positive cells by 52%, and by 51% of EYFP intensity in the remaining EYFP-positive cells at 1 μM compared to cells treated with dimethyl sulfoxide (DMSO) (Figure 5A). This reduction was similar to that observed by quantitative analysis of LSM images (Figure 5B). HUVEC cells showed a 93% reduction in the number of EYFP-positive cells and a 1.4-fold reduction in EYFP intensity in the remaining EYFP-positive cells at 1 μM (Figure S4B). A similar result was found by quantitative analysis of LSM images (Figure S4C).

Using the same experimental setup but with scAAV2-ANCH-mCherry, we investigated whether the reduction in protein expression was associated with a lower number of released genomes. Figure 5C shows a reduction to 34% of cells with any liberated genome in thapsigargin-treated cells. Correspondingly, the percentage of mCherry-expressing cells was reduced to 38% compared to the DMSO control. The reduction of marker protein–expressing cells was similar in U2OS (Figure S4D) and HUVEC cells, despite the use of different readouts (FACS, quantification after LSM).

To exclude that thapsigargin affected cytomegalovirus (CMV) promoter activity, OR-EGFP-U2OS cells were transfected with the plasmid bearing the ANCH-mCherry sequences. As shown in Figure 5D, mCherry-expression was not significantly different from the control after thapsigargin treatment. This was also observed in HuH-7 cells transfected with a plasmid expressing NLS-EGFP under CMV promoter control (Figure S4E). This observation suggests that thapsigargin’s effects are not primarily mediated by ER stress and stress granule induction, which can affect protein translation. Considering that thapsigargin did not abolish scAAV2 transduction in all cells, we investigated whether thapsigargin altered the Ca^2+^ distribution only in a fraction of cells. Therefore, we used the Ca^2+^ tracker Fluo-4FF, AM, in scAAV2-mCherry-transduced U2OS cells. As in the previous experiments, the percentage of transduced cells was reduced by 63% and the intensity of m-Cherry was reduced by 5.9-fold under thapsigargin treatment (Figure S4F, right panels). This was higher than the 2.04-fold reduction of scAAV2-EYFP-transduced cells. The higher sensitivity of FACS analysis compared to LSM may account for this difference, as the latter only allows the monitoring of cells with high fluorescence. In the DMSO control, Ca^++^ showed several foci outside the nuclei, presumably corresponding to the Ca^++^ reservoirs (Figure 5E). As was reported by others,^79^ thapsigargin treatment resulted in diffuse cytosolic Ca^++^ staining, which was not observed in the untreated cells and which indicates Ca^++^ relocation from the ER/Golgi. Ca^++^ redistribution was restricted to those cells that were refractory to thapsigargin-mediated inhibition of scAAV2-ANCH-mCherry transduction indicating differences in the cell population. The viability assay of the cells treated with the Ca^++^ tracker is shown in Figure S4G.

To distinguish whether thapsigargin affects viral capsid transport to the nucleus or genome liberation, we investigated the intracellular distribution of scAAV2-EYFP genomes. Cellular fractionation assays were performed using thapsigargin-treated cells. Treatment was started 1 h prior to transduction with 100 vg/cell for 2 h and maintained for 20 h when subcellular fractionation was performed. Purity of the fractions was verified by western blot using antibodies against histone 3, nucleoporins, and lamin A/C (Figure S4H). The distribution of the viral genomes, determined by ddPCR, was similar between the control DMSO and upon thapsigargin treatment (Figure 5F). This result indicates that thapsigargin and Ca^++^ did not affect scAAV2 trafficking from the cell periphery into the nucleus. For further confirmation, we visualized the capsids using A20 antibody by indirect immunofluorescence at 2 h p.t., While all cells in the DMSO-control showed nuclear capsids with an average of 17 copies, thapsigargin-treatment (Figure S4I), resulted in nuclear capsids in only 19% of the cells with an average of 2 capsids. In the remaining 81% of cells, capsids accumulated at the NE (Figure S4I), indicating that Ca^++^ is required for capsid entry into the nucleus.

To further investigate the effect of Ca^++^ on genome release, we next asked whether Ca^++^ induces structural changes in the capsid to allow access of nuclear proteins to the genome. We used the degradation of scAAV2-EYFP genomes by DNase I, determined by liquid droplet PCR, as marker. As positive control, we pre-treated capsids at 75^o^C, which has been shown to cause genome exposure of several parvoviruses.^80^ We observed that addition of Ca^++^ or of nucleoporins alone did not allow genome degradation (Figure 5G). However, in combination 66% of the genomes were degraded. In contrast, genome degradation could be blocked by the addition of WGA, suggesting that contact with O-GlcNAcylated sites of the nucleoporins is essential.

### Rad52, RPA1, and MRN complex act on genome release and degradation

**Figure 6:**
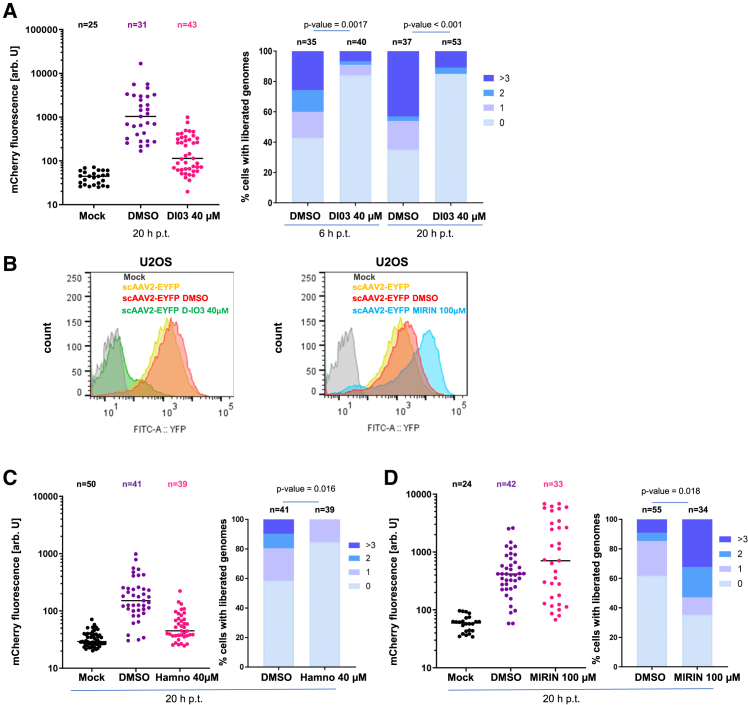
Effect of Rad52 and MRN on scAAV2 release (A) Left panel: quantification of mCherry expression in U2OS-OR-EGFP cells pre-treated with the Rad52 inhibitor D-I03 before scAAV2-ANCH-mCherry transduction (y axis, arb. U). The inhibitor treatment was continued during the following incubation of 20 h. n: number of analyzed cells is given on top of the figure and are derived from 7 images 20 h p.t. The bar is the median. Right panel: quantification of released scAAV2-ANCH-mCherry genomes in all cells at 6 h and 20 h p.t. (B) EYFP expression upon D-I03 (green, left panel), and Mirin (light blue, right panel) by FACS analysis 24 h post scAAV-EYFP transduction. The different conditions are given in different colors, which are described in the panels. (C) Left panel: mCherry expression upon HAMNO treatment quantified after LSM of 10 images. Y axis: mCherry fluorescence in arb. U. n: number of analyzed cells. Right panel: quantification of released scAAV2-ANCH-mCherry genomes. For legend see (A). (D) as in (C) but upon Mirin treatment.

The observation that Ca^++^ exposure triggered genome exposure implied that genome release within the nucleus may occur via interaction with the ITRs, presumably requiring interaction with DNA-damage response proteins. We therefore transduced OR-EGFP expressing U2OS cells with scAAV2-ANCH-mCherry using inhibitors of Rad52 and Mre11. Using LSM, we observed that D-I03 inhibiting Rad52-decamerization resulted in a 10-fold reduction of mCherry expression, which was confirmed by FACS analysis of the cells transduced with scAAV2-EYFP (Figure 6A left panel, 6B left panel). We excluded an inhibitory effect on protein expression in cells transfected with the genome-containing plasmid (Figure S5A, right panel). Investigating the effect of D-I03 on genome release, we observed a reduction to 50% of cells with released genomes compared to the control (DMSO) at non-toxic D-I03 concentrations (40 μM; Figure S5A, left panel), and at 6 h and 20 h p.t., respectively (Figure 6A, right panel).

Considering that Rad52 decamerization is triggered by the RPA complex, we investigated if the inhibition of Rad52-RPA complex formation was required. We used HAMNO, which binds to the N-terminus of RPA70, blocking RPA32 phosphorylation,^63^ which in turn is required for Rad52 interaction with the RPA complex triggering Rad52 decamerization. Adding this inhibitor to scAAV2-ANCH-mCherry-transduced cells, we observed significant inhibition of the percentage of mCherry-expressing cells by 70% at 20 h p.t. (Figure 6C, left panel) but also of genome release, both compared to the DMSO control (Figure 6C, right panel). At the applied concentration, no toxicity could be observed (Figure S5B, left panel), and mCherry expression after plasmid transfection was not impaired (Figure S5B, right panel).

Analyzing the effect of Mirin, an inhibitor of the exonuclease activity of Mre11,^81^ we observed the opposite effect. The inhibitor stimulated EYFP expression by 3.8-fold in cells transduced with scAAV2-EYFP analyzed by FACS, but also by scAAV2-ANCH-mCherry transduction and LSM analysis (Figure 6B, middle panel; 6D, left panel). The expression was not affected in cells transfected with the genome-containing plasmid (Figure S5C, right panel). The increase in EYFP/mCherry expression corresponded to a 56% increase in released genomes in scAAV2-ANCH-mCherry-transduced cells (Figure 6D, right panel).

The inhibitory effect of Mre11 led us to ask whether this protein is involved in the disappearance of scAAV2 genomes after reaching a plateau. We quantified the genomes in isolated nuclei of 1 h scAAV2-EYFP-transduced Mirin-treated U2OS cells by digital PCR. Figure S5Dshows a similar result of transduced cells with and without DMSO. The numbers of nuclear viral genomes at 8 h was 2.3-fold higher than at 24 h (Mirin), suggesting that Mre11 activity accounted for degradation of released scAAV genomes during the time course of infection. Considering the effects of Rad52 and Mre11 we investigated their potential co-localization with scAAV2 genomes. Figure S6 shows a diffuse intracellular localization indicating that only a part is oligomerized in U2OS cells. Nuclear Rad52 showed that the scAAV2 genomes are located in the vicinity of Rad52 and Mre11, some of which show partial or complete co-localization (Figures S6A–S6D). However, not all scAAV2 genomes perfectly co-localized, which can be interpreted in that interaction with Rad52 is a temporal process occurring just during genome release, and only as long as the genome is not circularized.

Non-transduced cells show similar distribution of Rad52 and Mre11 (Figure S6C). Furthermore, the genomes were located at the boundary between condensed and non-condensed chromatin.

## Discussion

scAAVs are the simplest form of non-replicating vectors, consisting of three coat proteins and an artificial DNA flanked by ITR. This simplicity enables the investigation of infection without interference from viral replication and gene products.

### Kinetics of ANCHOR system-based genome detection

Our analyses to understand the mechanism of scAAV genome release were primarily based on time-lapse microscopy, using scAAV2 bearing the anchor sequence, which serves as the initiation point for OR-EGFP accumulation. The specificity of the signals was demonstrated by their occurrence exclusively in transduced cells, with mCherry expression limited to cells displaying genome signals. During interphase, genome signals were confined to the nuclei, consistent with a nuclear genome release. These signals, in line with the turnover of OR-EGFP after ANCH-binding,^66^ exhibited resistance to bleaching, a characteristic not observed for OR-EGFP clusters, facilitating their discrimination.

Foci derived from released genomes showed variation in both intensity and size. Nevertheless, when normalized to intra-nuclear OR-EGFP expression, the intensity was homogeneous. This suggests that increased signal strength did not result from genome clustering, but rather from the number of OR-EGFP molecules associated with each ANCH. In support of this interpretation, the signal intensity remained constant over the course of several hours. While the size of the foci displayed variability, we observed a similar distribution pattern following microinjection of plasmid DNA bearing ANCH, indicating that the signals were originated from individual isolated DNA molecules. Consequently, we conclude that we observed individual scAAV2-ANCH-mCherry genomes, and the variation in size reflect different organizational forms of the DNA rather than dimers or multimers. According to this hypothesis, the number of foci corresponds to the number of capsid-released genomes, aligning with findings related to human cytomegalovirus genomes labeled using a similar technique.^82^

Microinjection of ANCH plasmid DNA provided further confirmation that the fluorescent system allows rapid detection of the released genomes. This kinetic of foci development was just technically limited by the time it takes to switch between microinjection and microscope imaging modes, which is approximately 30 s. These findings suggest that the appearance of released genomes could be monitored practically in real-time. Transduction experiments revealed that full signal strength was achieved at least within 10 min after the first genome was detected, indicating that the release is rapid and results in complete genome exposure.

### Kinetics of scAAV transduction

Analyzing the number of released genomes, we observed a wide range of copies after 24 h, with a median of 3 h, which was the same by microscopy and ddPCR. The copy number differed significantly from the 19 genomes found in the nucleus by ddPCR and from the 20 nuclear capsids, identified by indirect immunofluorescence. This finding suggests that only approximately 10% of capsids released their genomes during the observation period, indicating that this step is the rate-limiting factor within the cell.

Time-lapse microscopy revealed the presence of released scAAV2-ANCH-mCherry genomes as early as 2 h p.t. This time is shorter than the 3 h observed by Cervelli et al., but this study did not determined genome release but the time taken until conversation of AAV2 with a ss genome to dsDNA.^48^ Similar to their observation of a maximum accumulation at approximately 8 h post infection, we observed a maximum number of released genomes at an average of 11 h in cells in which an observable genome degradation occurred. Nevertheless, the appearance of scAAV2-ANCH-mCherry genomes was variable, requiring further investigations of differences between individual cells for interpretation. We also observed intercellular differences in thapsigargin sensitivity and mCherry expression, with the latter being inherited by daughter cells during cell division, suggesting genetic variability within the U2OS cell line.

### Events at the NE

Our data revealed no genome release at the NE, in contrast to other DNA and retroviruses studied to date. Instead, scAAV2 genome release occurred deeper in the nucleus, suggesting a previously unreported strategy.

By *in vitro* experiments, we observed that genome release is initiated by limited capsid opening, which occurs upon simultaneous contact with glycosylated nucleoporins and exposure to Ca^++^ concentrations found in the ER/NE, which is higher in this compartments.^83^ However, opening did not result in genome release, arguing against the hypothesis of AAV genome ejection proposed by others using atomic force microscopy.^50^ Instead, our findings support the idea that the capsid, once opened but otherwise intact, enters the nucleus, where it reaches the release site. This suggests that capsids entering the nucleus to initiate infection are derived from these Ca^++^-exposed capsids bound to the NPC, rather than from those that entered the nucleus through holes created by Ca^++^ efflux during NE permeabilization.^47^ This hypothesis is further supported by our finding that thapsigargin treatment resulted in capsid accumulation at the NE and prevented nuclear capsid entry.

Although our study has not investigated the detachment of the capsids from the NPC, we hypothesize that the AAV2 capsids dissociate together with isolated Nups as calcium signaling can trigger the heterogeneous dissociation of nucleoporins from NPCs, which includes Nup62, Nup153, and Nup214^84^ to which parvoviral capsids bind.^47^ This scenario is in agreement with findings of Mantyla et al. who observed Nup153 associated with nuclear CPV capsids.^33^

While Ca^++^-dependent conformational changes of proteins are well-documented, often resulting in stabilization,^85^ the knowledge of their impact on viral capsids is limited. However, two polyomaviruses, namely Simian virus 40 and Merkel cell polyoma virus, require exposure to elevated Ca^++^ levels as they traverse the endolysosomal network, from which they escape into the cytoplasm and enter the nucleus.^86^^,^^87^^,^^88^^,^^89^ Evidently, this pathway differs from that what we observed in the case of scAAV2.

Our findings contrast with recent observations by Sutter et al. (2022),^90^ who reported that ss recombinant AAV genome release occurs within nucleoli.^90^ In our study, we observed released viral genomes exclusively at the cellular chromatin, as demonstrated by co-localization in live cells, after permeabilization by digitonin, and through subcellular fractionation. Notably, Sutter et al. employed 200-fold higher viral concentrations in their experiments, suggesting that distinct mechanisms may be involved at different viral loads or a different pathway for scAAV. This hypothesis is supported by the conclusions of others proposing a model in which the nucleolus is a reservoir for incoming AAV from which they are sequestered from the cytosol.^91^

### Intra-nuclear events

In contrast to other viruses, the scAAV2 genomes did not co-localize at or near PML nuclear bodies as it was observed for e.g., adenoviruses, HSV1 and papovaviruses.^92^ Instead, the scAAV2-ANCH mCherry genomes co-localized or localized in the vicinity of Rad52 and Mre11 and were arrested close to or at chromatin.

While the localization at Rad52 and Mre11 is consistent with their roles in genome release and degradation, the latter observation aligns with the spatial arrest of the genomes, as reported by others.^48^ However, in contrast to results of others using wt MVM, we can exclude an NS1-mediated directed transport to sites of DNA damage where Rad52 and Mre11 accumulate,^93^ since our scAAV2 lacks the NS1 homologue rep78.

Our focus on Rad52, Mre11 (along with the Rad52 activity-modulating RPA) was guided by prior observations that demonstrated interactions between Rad52 and Mre11 with the AAV genome.^48^^,^^52^^,^^53^ If Ca^++^/NPC interactions are responsible for opening the capsid, we hypothesized that the ITR on nuclear AAV capsids would be accessible prior to genome release. Considering that Rad52 acts not as a monomeric protein but as a homedecamer,^56^ we could address its function not by CRISPR/cas9 knock-out experiments but by inhibitors. In turn, toxicity of the inhibitors restricted their concentration, hardly allowing complete inhibition.

We observed that inhibition of Rad52 directly by DI03 or indirectly, disrupting the Rad52-RPA complex formation by HAMNO^63^ resulted in a similarly strong reduction in genome release, suggesting that its supportive role in genome replication occurs early in the process. Furthermore, this finding is consistent with scAAV2 genome localization in the vicinity of chromatin, where Rad52 decamerization is likely triggered by binding to the RPA complex.^56^^,^^60^^,^^94^ Furthermore, we cannot assume that all released viral genomes are permanently associated with Rad52, but only during the process of genome release, and this could be dynamic, making it difficult to achieve accurate colocalization.

Surprisingly, we observed that scAAV2-ANCH-mCherry genomes became randomly distributed during cell division following transduction. This finding suggests that the binding to chromatin is disrupted upon cell division. One potential pathway explaining this observation is the displacement of RPA from single-stranded DNA, a process that occurs when cells enter the S – M phase transition, facilitated by topoisomerase II.^95^

Mre11 may have acted as a counterpart to Rad52. We observed that its inhibition not only increased the number of scAAV2-ANCH-mCherry genomes released in 66% of cells but also prevented their subsequent disappearance. This observation aligns with previous findings, which demonstrated that silencing of Nbs1 in the MRN complex prevented the disappearance of rAAV2 genome.^48^ Furthermore, productive wtAAV infection is impeded by adenoviral co-infection, as the adenoviral E1b55K/E4orf6 proteins induce degradation of the cellular Mre11.^53^

As such, we interpret the finding that 23% of cells exhibited a sustained increase in released genomes as an indication of potential alterations or delays in the expression of MRN components, considering that degradation may also occur after the observation period.

**Figure 7:**
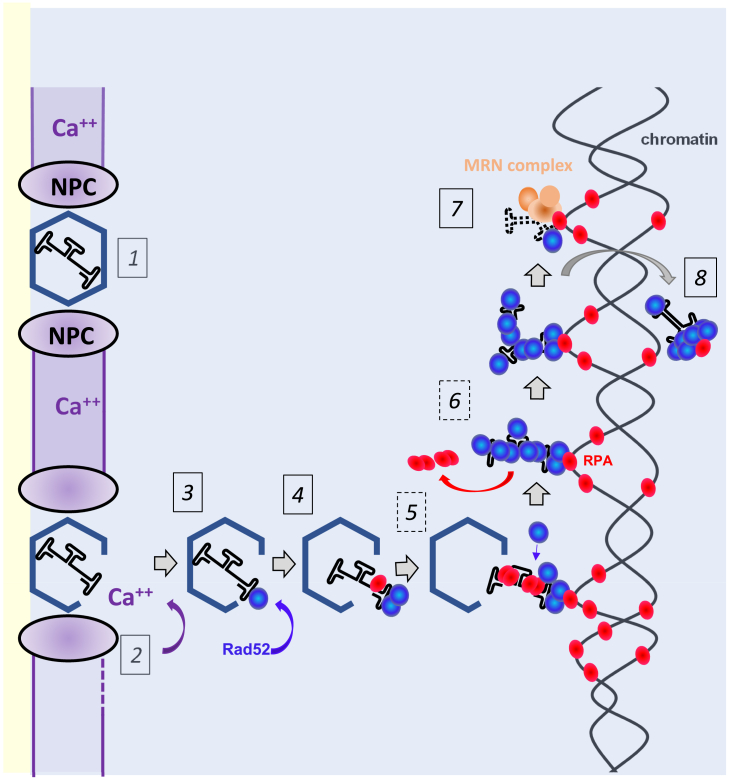
Schematic presentation of scAAV2 fate at the NE and within the nucleus **1.** Capsid binding to the NPC via importin β. **2.** Ca^++^ exposure derived from the lumen between inner and outer nuclear membrane opens the capsids while being in contact to glycosylated proteins of the NPC. **3.** Capsid release into the nucleus. **4.** Rad52 recruitment to the exposed scAAV genome. **5.** Attachment to the host chromatin probably mediated via RPA Rad52-binding. **6.** Rad52 RPA interaction probably leads to decamerization of Rad52 causing genome release. These 5 and 6 steps are shown as discontinuous square due to the indirect evidence for the role of RPA shown in this work. **7.** Recruitment of the MRN complex, which competes with Rad52-binding causing scAAV2 genome degradation. **8.** Release of scAAV genome from chromatin during mitosis, potentially caused by topoisomerase II activity.

Although we admit that functionally analogous pathways may participate in scAAV2 genome release, our work has provided evidence that scAAVs employ an unknown strategy for genome release. This seems to be similar to regular vectors according with the time frame of double-stranded genome formation observed by Cervelli et al.^48^ and appears to be the key limiting factor in transduction; this finding may offer opportunities to improve gene therapy (Figure 7).

Our findings allow to propose a model of scAAV2 capsid nuclear import and genome release, which can be potentially transposed also to other parvoviruses. Capsid interaction with the NPC initiates structural changes that result in Ca^++^ efflux from the NE. Our model suggests that only those capsids that interact with the NPC and are exposed to the high calcium concentrations between the inner and outer nuclear membranes undergo structural changes, leading to their release from the NPC and to exposure of the ITRs. These Ca^++^-induced capsid alterations – which might be indirectly mediated via nucleoporins – result in capsids, which differ from progeny capsids assembled in the nucleus, explaining why the latter, which do not expose their ITRs, remain intact.

ITR exposure could then allow interaction with Rad52 which, as described in another process, upon engagement with the RPA complex, could form decamers in the vicinity of host chromatin, leading to complete genome release. The released genome is subsequently degraded by the MRN complex, possibly after Rad52 degradation or translocation, resulting in the gradual disappearance of the scAAV genomes. While Mirin inhibits the nuclease activity of Mre11, consistent with our hypothesis of genome degradation, we cannot exclude the possibility that the observed increase in liberated genomic arises from the accumulation of DNA damage under Mirin treatment, by the inhibition of ATM or the disruption of the G2 checkpoint. Following our hypothesis, our data suggest that temporary treatment with an Mre11 inhibitor, or drugs targeting other components of the MRN complex not evaluated in our study, could substantially enhance scAAV2 transduction or improve the efficiency of oncolytic parvoviruses. The feasibility of such an approach is demonstrated by the use of Mirin as a sensitizer, which enhances radiosensitivity in glioma therapy.^96^

### Limitations of the study

scAAV Genome Release Mechanisms at Different Viral Loads: Nuclear scAAV genome release contrasts with nucleolar release at high viral loads, suggesting load-dependent mechanisms. Factors like receptor saturation, packaging, and stability might contribute. High loads can also increase toxicity and immune responses, affecting release.

Rad52 Association with Released Viral Genomes: Co-localization of released genomes with Rad52 and Mre11 suggests their involvement in genome processing. Rad52, important for DNA repair and integration, may transiently associate during release or early processing. Thus, live-cell co-localization studies face technical limitations.

Chromatin Binding Disruption During Cell Division: Random viral genome distribution during cell division indicates disrupted chromatin binding. RPA likely initiates binding, and cell cycle-dependent changes in RPA and enzymes like topoisomerase II could cause displacement.

MRN Complex Component Alterations and Genome Release: The MRN complex degrades released scAAV genomes. Sustained genomes increase in some cells may result from variations in MRN component expression, potentially due to cellular genetic variability. MRE11 inhibition leads to DNA damage, supporting its role in degrading foreign DNA.

Therapeutic Potential and Safety of Targeting the MRN Complex: Temporary Mre11 inhibition could enhance scAAV transduction. However, MRE11’s role in DNA repair requires careful consideration of side effects like DNA damage and genomic instability. Interactions with other therapies and cell metabolism may influence outcomes. Off-target effects and inhibition duration need careful control.

### Perspectives

The study provides valuable insights into scAAV transduction, but its limitations require further investigation. Future research could use single-cell sequencing for investigating differences between individual cells, explore the role of calcium in capsid destabilization and structure, compare mechanisms at different viral loads and in synchronized cells, demonstrate protein interactions by live-cell imaging, elucidate the disruption of chromatin binding during cell division, analyze the regulation of the MRN complex, and evaluate the safety of MRN complex inhibition for therapeutic applications. Continued research in these areas is critical to the advancement of gene therapy and oncolytic virology. A system to track uncoating in combination with the scAAV anchor system would allow uncoating and genome release to be observed simultaneously in individual living cells.

## Resource availability

### Lead contact

Further information and requests of resources and reagents should be directed to the lead contact, Michael Kann (Michael.kann@gu.se).

### Materials availability

Antibodies were obtained from commercial sources described in the STAR Methods key resources table. All unique materials generated in this study will be available from the lead contact after completing a Material Transfer Agreement.

### Data and code availability

• Data:
  ○ **ID: FR-FCM-Z6XB**, Analysis of scAAV2 transduction in U20S under Thapsigarging treatment. http://flowrepository.org/id/RvFrv5RHbIuZqiB6xv428BYm2ajh8rsPCWvFXPUciMfURwGj5YlxmOBL2Se6Udb1.
  ○ **ID: FR-FCM-Z6XC**, Analysis of scAAV2-YFP transduction in HUVEC cells under Thapsigargin treatment. http://flowrepository.org/id/RvFrcvDsuwldqHAVLlRrZEWgbCVFG5ELmxnriL3oUQls1Zuq0pE4cAtDGgAAEX88.
  ○ **ID: FR-FCM-Z6XD**, Analysis of scAAV2-YFP transduction in U2OS under repair protein inhibitors; D-I03 (Rad52 inhibitor), MIRIN (Mre11 inhibitor). http://flowrepository.org/id/RvFriKZtVc77WH2EQpdKvpXSI2fg6fIRlMsniNpu74Qk03BHkMmUgxDoHS5FLhJG.
  ○ Bustamante Jaramillo, Luisa (2025), “Manuscrito scAAV2 genome”, Mendeley Data, V1, https://doi.org/10.17632/bv253dc4rb.1.
• Code: This paper does not report original code.

## Acknowledgments

The work was supported by a start-up grant from the 10.13039/501100005760University of Gothenburg to M.K. We also thank the Center for Cellular Imaging Facility (CCI) at the University of Gothenburg and Lill Mårtensson-Bopp at the Department of Rheumatology at Gothenburg University for training and the use of the microscopy facilities. We thank the members of Kristoffer Hellstrand's group and the TIMM Laboratory (Hanna Grauers Wiktorin, Junko Johansson and Chiara Badami) for their support and access to the flow cytometry machines at the Salgrenska Center for Cancer Research. Finally, we thank the students and researchers who stayed in the laboratory for training (Lina Andersson, Laura Sanz Asensio, Edoardo Pizzioli and Daniel A. Bustamante Jaramillo).

## Author contributions

L.F.B.-J., J.F., L.Y., G.R., and M.J. conducted the experiments; L.F.B.-J. and M.K. designed the experiments and wrote the paper. L.F.B.-J., J.F., T.E., and M.K. analyzed the data. M.-L.B. and F.G. constructed the scAAV2-ANCH-mCherry vector; L.F.B.-J., O.M., S.H., M.M., and Q.C. constructed the scAAV2-EYFP vector and assisted and/or performed the expression and purification. All authors participated in the revision of the manuscript.

## Declaration of interests

F.G. is founder and shareholder of NeoVirTech, which holds the patent of the anchor system. All other authors declare no competing interests.

## STAR★Methods

### Key resources table

**Table:** 

REAGENT or RESOURCE	SOURCE	IDENTIFIER
**Antibodies**		

Rad52 monoclonal	Invitrogen	Cat# MA5-31888; RRID: AB_2787511
Mre11 monoclonal	Abcam	Cat# ab214; RRID: AB_302859
Goat anti-mouse igG (H+L) highly cross-absorbed secondary, Alexa Fluor plus 647	Invitrogen	RRID: AB_2535805
A20	Müller Laboratories	
Histone H3 (D1H2) XP® rabbit mAb		Cat# 4499; RRID: AB_10544537
Nucleoporins		Abcam Cat# ab24609; RRID: AB_448181
Lamin A/C conjugated with horseradish peroxidase (HRP)		Cat# NBP1-97688H; RRID: AB_3243320
Rabbit anti-mouse HRP	DAKO	PO260

**Bacterial and virus strains**		

scAAV2-ANCH-mCherry	Neo Vir Tech SAS	
scAAV2-YFP	Müller Laboratories	
scAAV2-mCherry	Müller Laboratories	

**Chemicals, peptides, and recombinant proteins**		

DMEM	Thermo Scientific	Cat.No. 41965039
Endothelial Cell Growth Medium 2	PromoCell	Cat, No. C-22211
Endothelial Cell Growth Medium 2 KIT	PromoCell	Cat, No. C-22111
PEST (10,000 Units/10,000 μg/mL)	Thermo Scientific	Cat.No. 15140122
Fluo-4FF, AM	Invitrogen	F23981
Thapsigargin	Invitrogen	T7459
HAMNO	Sigma-Aldrich	SML1234
D-I03	Sigma-Aldrich	SML2496
Hygromycin B (50 mg/mL)	Thermo Fisher	Cat.No. 15140122
PEI (25 kD linear)	Polysciences	Cat. No. 23966
TurboFect	Thermo Fisher	Cat. No. R0531
Benzonase	Sigma Aldrich	Cat.No.E1014-25KU
Optiprep™	Sigma Aldrich	Cat. No. D1556
Phenol red solution	Sigma Aldrich	Cat. No. P0290
Hoechst 33342 (20 mM)	Thermo Fisher	Cat. No 62249

**Critical commercial assays**		

MagNA Pure LC DNA isolation kit	Roche	
CellTiter 96, One solution, Cell Proliferation Assay	Promega	G3580
Subcellular Protein Fractionation Kit for Cultured Cells	Thermo Fisher	78840

**Deposited data**		

Analysis of scAAV2 transduction in U20S under Thapsigarging treatment	http://flowrepository.org/id/RvFrv5RHbIuZqiB6xv428BYm2ajh8rsPCWvFXPUciMfURwGj5YlxmOBL2Se6Udb1	ID: FR-FCM-Z6XB
Analysis of scAAV2-YFP transduction in HUVEC cells under Thapsigargin treatment	http://flowrepository.org/id/RvFrcvDsuwldqHAVLlRrZEWgbCVFG5ELmxnriL3oUQls1Zuq0pE4cAtDGgAAEX88	ID: FR-FCM-Z6XC
Analysis of scAAV2-YFP transduction in U2OS under repair protein inhibitors; D-I03 (Rad52 inhibitor), MIRIN (Mre11 inhibitor)	http://flowrepository.org/id/RvFriKZtVc77WH2EQpdKvpXSI2fg6fIRlMsniNpu74Qk03BHkMmUgxDoHS5FLhJG	ID: FR-FCM-Z6XD
Mendeley Data, V1, Manuscrito scAAV2 genome	https://data.mendeley.com/datasets/bv253dc4rb/1	https://doi.org/10.17632/bv253dc4rb.1

**Experimental models: Cell lines**		

HEK293TT cells	Müller Laboratories	
HUVEC cells	Department of infectious diseases. Gothenburg University (GU)	
U20S	Department of infectious diseases. GU	
U20S-OR-GFP	This paper	

**Oligonucleotides**		

β-globin. forward primer: GCTCATGGCAAGA AAGTGCTC; reverse primer, GCAAAGGTGC CCTTGAGGT; Probe: AGTGATGGCCTGGC TCACCT GGAC.		
CMV forward primer (CMV enhancer 329F): TGC CCA GTA CAT GAC CTT ATG G CMV; Reverse Primer (CMV Enhancer 462R): GAA ATC CCC GTG AGT CAA ACC CMV; Probe (CMV Enhancer 383T): 6-FAM AGT CAT CGC TAT TAC CAT GG -TAM.		

**Recombinant DNA**		

scAAV2-ANCH-mCherry plasmid	Neo Vir Tech SAS	
scAAV2-YFP plasmid	Müller Laboratories	
Helper plasmid	Müller Laboratories	
Capsid plasmid	Müller Laboratories	

**Software and algorithms**		

Image J (Fiji)	Gothenburg university (GU)	https://imagej.nih.gov/ij/
Zeiss zen 3.2 blue	Centre for cellular imaging (CCI). GU	https://www.zeiss.com/microscopy/int/products/microscope-software/zen-lite.html
CellProfiler	Centre for cellular imaging (CCI). GU	https://cellprofiler.org/
Graphpad	GU license	
Image Lab	GU license	

**Other**		

Slide 8 well	Ibidi	80826
Eppendorf FemtoJet 4 and Injectman N12 coupled to a Zeiss Observer Z1 microscope.	Eppendorf	
μ-slide VI 0.1.	Ibidi	

### Experimental model and study participant details

**HEK293TT cells** were grown in DMEM (Gibco™)/10 % heat-inactivated FCS/2 % L-glutamine/1 % penicillin-streptomycin (PEST)/1.25 % hygromycin B. **HUVEC cells** were grown in Endothelial Cell Growth Medium (MEDIQIP C-22111, C-22211), and **OR-EGFP U2OS or U20S cells** were grown in DMEM/5 % FCS/1 % PEST. Cells were maintained by routine splitting when they reached ∼70-80 % confluence. All cell lines were tested for mycoplasma contamination by PCR. The cell lines have not been authenticated.

### Method details

#### scAAVs production

scAAVs stocks were prepared based on two published methods,^97^^,^^98^ following the so-called three-plasmid system. scAAVs were extracted from HEK293TT transfected cells by cell lysis (150 mM NaCl, 50 mM Tris-HCl, pH 8.5) and 5 cycles of freezing and thawing. The solution was then treated with 250U benzonase and incubated at 37°C for 30 min. Viruses were purified by iodixanol (Optiprep™ Sigma Aldrich) gradient centrifugation normally yielding in 4 x 10^7^ vg, which was insufficient for *in vitro* labelling.

#### Droplet digital PCR

Viral genome extraction was performed using MagNA Pure LC DNA Isolation Kit (Roche) on an automated MagNA Pure LC 2.0 instrument, and droplet digital PCR (Bio-Rad) was performed using the following primers and probe: CMV forward primer (CMV enhancer 329F): TGC CCA GTA CAT GAC CTT ATG G CMV; Reverse Primer (CMV Enhancer 462R): GAA ATC CCC GTG AGT CAA ACC CMV; Probe (CMV Enhancer 383T): 6-FAM AGT CAT CGC TAT TAC CAT GG -TAM. The Bio-Rad Automated Droplet Generator was used, which produces around 20,000 nanolitre droplets using oil as a separator. The droplet samples in the plate are then transferred to an Applied Biosystems® Veriti® 96-well thermal cycler where the PCR is performed. The composition of the reaction mix and the heat profile was described elsewhere.^99^ Quantitative readout of the samples was determined using the Bio-Rad QX200™ Droplet Reader. When required, β-globin was used as the target for cell number normalisation. The forward primer was GCTCATGGCAAGAAAGTGCTC; the reverse primer was GCAAAGGTGCCCTTGAGGT; and the probe was AGTGATGGCCTGGCTCACCT GGAC.

#### Inhibitor treatment

3 x 10^3^ U2OS-OR-EGFP or U2OS cells were seeded in μ-slide VI 0.1 channels (Ibidi, 80666). The cells were pre-treated for 1-2 h with thapsigargin (Invitrogen, T7459) 1 μM, Mirin (Sigma-Aldrich, M9948), HAMNO 40 μM (Sigma-Aldrich, SML1234), or 3 h for D-I03 (Sigma-Aldrich, SML2496) 40 μM. After pre-treatment, the cells were transduced with 100 vg/cells of scAAV2-ANCH-mCherry or scAAV2-EYFP for 1 h in DMEM with the inhibitor. After 20 h in the presence of the inhibitor, the cells were placed on the LSM700 confocal microscope at 5 % CO_2_, 100 % humidity and 37°C. For FACS analysis, the treatment was similar but 2.5 x 10^4^ U2OS cells were seeded in 24-well plates and after trypsination, were analysed with LSR-Fortessa X-20. HUVEC cells were transduced with 1000 vg scAAV2-EYFP/cell for 24 h.

For transfection, 1.7 x 10 ^4^ U2OS-OR-EGFP cells were seeded in Slide 8 well (Ibidi, 80826). One day later, the cells were transfected with 50ng/μl of scAAV2-ANCH-mCherry plasmid using 0.5 μl of TurboFect (Thermo Fisher, R0531), and following the manufacturers’ instructions. 24 h later, the medium was changed, and the cells were treated with the corresponding inhibitor for 24 h and analysed by LSM.

#### Monitoring of intracellular Ca^++^

U2OS cells were pre-treated with thapsigargin 1 μM for 2 h, followed by transduction with scAAV2-mCherry for 1 h, and then maintained in DMEM-thapsigargin for 20 h. After 20 h the medium was changed and medium with 1 μM Fluo-4FF, AM, (Invitrogen, F23981) was added for 30 min. The medium was then changed to medium containing 400 ng/ml Hoechst 33342 (Thermo Fisher) and incubated for a further 30 min before time-lapse microscopy at 5 % CO_2_, humidity and 37°C.

#### Viability assay

5 x 10^3^ cells were seeded in 96-well plate. One day later, the cells were treated with Thapsigargin 0.01, 0.025, 0.05, 1 μM/D-I03 20, 40, 80 μM/Mirin 80, 100, 200 μM/HAMNO 20, 40, 80 μM and Fluo4-FF, AM 1,2,4 μM for 24 h. Each concentration was assayed in triplicate, and DMSO was used as control. The viable cells were then analysed using CellTiter 96, One solution, Cell Proliferation Assay (Promega G3580) according to vendor’s protocol. The absorbance was measured using the 96-well plate reader Multiskan FC (Thermo scientific).

#### Microscopy and image analysis

3 x 10^3^ U2OS-OR-EGFP were seeded in μ-slide VI 0.1. 24 h later, the cells were transduced with the scAAV2-ANCHOR-mCherry overnight or 1 h as indicated in the results. After 24 h the cells analysed by time-lapse microscopy using a Confocal LSM700. During microscopy the cells were kept at 5 % CO_2_, H_2_O saturation at 37°C. Images were taken as z-series with 9 optical sections of a total of 8 μm. Images were taken every 10, 30 min or 1 h for 24 or 36 h. Images were analysed using Image J (Fiji), Zeiss zen 3.2 blue and/or CellProfiler. The kinetics of released genomes was analysed by counting the genomes in each slide from each z-series at each time point in different cells from independent experiments. To analyse the mobility of the genomes, the distance of the genomes to the NE and to the centre of the nucleus at each time point was obtained from CellProfiler. Quantification of released genomes was performed by manually analysing each slide and frame. Four categories were established; cells with 0, 1, 2 and more than 3 liberated genomes. Statistically significances were calculated by comparing inhibitor-treated cells with the controls (DMSO) using the chi-square test (qualitative data) and Mann-Whitney-U test (quantitative data, two groups of data) or Kruskal-Wallis test (quantitative data, 3 or more data groups). To calculate the reduction in the percentage of cells with any liberated genomes, the percentage of DMSO-treated cells with 0 liberated genomes was subtracted from the percentage of inhibitor-treated cells with 0 liberated genomes. To calculate the reduction of the percentage of mCherry expressing cells by LSM, the MOCK control was used to establish the threshold of negative mCherry expressing cells. The percentage of positive mCherry expressing cells was calculated for DMSO and inhibitor-treated cells. The correction for positive inhibitor-treated cells was performed by considering the percentage of positive cells in DMSO as 100 %. The percentage of negative inhibitor-treated cells was subtracted from 100 minus the percentage of positive inhibitor-treated cells after correction. Graphics and statistics were performed using GraphPad Prism 7. Quantitative parameters were compared using the Mann-Whitney U test for significant differences.

#### Localisation of scAAV2 genomes, capsids and host factors

For transfection, U2OS-OR-EGFP cells were seeded in Slide 8 well (Ibidi, 80826). One day later, the cells were transfected with PML and USP7-fusion protein-expressing plasmids using TurboFect (Thermo Fisher, R0531), according to the manufacturers’ instructions. 24 h later, the cells were transduced with scAAV2-ANCH-mCherry for 24 h and analysed by time-lapse microscopy.

For immunofluorescence analyses, U2OS-OR-EGFP were seeded in μ-slide VI 0.1, and transduced with scAAV2-ANCH-mCherry for 24 h. Cells were washed in PBS prior to fixation using Psuc buffer (PFA 4 %/sucrose 2 %) for 3 min at RT, followed by 3 min in -80°C methanol/acetone. Cells were then washed twice with PBS. Inmunostaining was performed with A20 antibody 1/200, Rad52 monoclonal antibody (Invitrogen, MA5-31888) 1/200 or Mre11 monoclonal antibody (Abcam, Ab214) 1/200 and Goat anti-mouse IgG (H+L) highly cross-absorbed secondary antibody, Alexa Fluor™ plus 647 (Invitrogen) 1/2000 according to the manufacturer. DAPI (Sigma Aldrich) was added at 1 μg/ml in PBS.

The quantification of the colocalization between scAAV2 genomes and Rad52 showed in Figure S6D was performed using Manders' overlap coefficient. This coefficient measures the fraction of the intensity of one channel (scAAV2) that overlaps with the structures in the other channel (Rad52). A value close to 1 indicates a high degree of overlap, while a value close to 0 suggests little or no overlap. This analysis was conducted using the JACoP plugin within ImageJ. (Bolte et al., 2006. A guided tour into subcellular colocalization analysis in light microscopy. *Journal of Microscopy*, *224*(3), 213–232. https://doi.org/10.1111/j.1365-2818.2006.01706.x). Each value was calculated within defined regions of interest to focus on a single scAAV2 region. Images were corrected with a smooth filter to ease the selection of the thresholds. To assess the significance of the measured colocalization, a randomization analysis was performed. The foci of the scAAV2 genomes were shifted within the image according to defined quadrants, and the Manders' overlap coefficient was recalculated for each randomized position. This approach allowed us to compare the colocalization values observed in our samples with a distribution expected by chance.

#### Isolation of nuclei

1.5 x 10^5^ U2OS cells were seeded in M24 plates and cultured at 37°C for 24 h. After scAAV2-EYFP transduction and/or inhibitor treatment, the cells were treated with 4.2 μM cytochalasin B (C6762)/DMEM for 30 min at 37°C. After trypsination, the cells were collected in 3 ml PBS and centrifuged at 200 x g for 10 min at 4 °C. Washing and centrifugation steps were repeated 2 times. Cells were resuspended in 1 ml nuclei buffer (10 mM Pipes/10 mM KCl/2 mM MgCl_2_/1 mM DTT) and centrifuged as before. Cells were resuspended in 10 volumes of the pellet in nuclei buffer containing 10 μM cytochalasin B and incubated for 30 min on ice. The cells were then homogenised with 30 strokes using a Dounce homogeniser on ice, and the sample was added to 4 volumes of 30 % (w/w) sucrose/nuclei buffer and centrifuged at 800 x g and 4°C for 10 min. The pellet was resuspended in 200 μl of nuclei buffer, the centrifugation step was repeated, and the pellet was suspended in 100 μl of nuclei buffer. The scAAV2-EYFP genomes in the samples were extracted and quantified by droplet digital PCR as previously described. Normalisation for the number of isolated nuclei was performed using β-globin.

#### Extraction of nucleoporins

Nucleoporins were extracted according to the protocol of Matunis.^100^ 3 x 10^6^ frozen nuclei and thawed. Insoluble components were sedimented for 1 min at 200 x g at 4°C. The pellet was suspended in 0.1 mM MgCl_2_/5 μg/ml DNase I/5 μg/ml RNase A/ 1 mM DTT/1x protease inhibitor mix and 4 ml extraction buffer pH 8.5 (10 % sucrose [w/v] /20 mM triethanolamin pH 8.5/1 mM MgCl_2_ /1 mM DTT) was added, following incubation for 15 min at RT. The lysate was added to 4 ml of an ice-cold cushion of 30 % sucrose (w/v)/20 mM Triethanolamin pH 7.5/ 0.1 mM MgCl_2_/1 mM DTT/1x protease inhibitor mix and centrifuged for 15 min at 4°C and 4000 x g. The sediment was suspended in 1 ml extraction buffer (pH 7.5) and mixed with 0.5 ml 0.3 mg Heparin/ml extraction buffer (pH 7.5). The sample was sedimented for 15 min at 4°C and 4000 x g through a 4 ml sucrose cushion for removal of chromatin and ribosomes. The pellet was suspended in 1 mL extraction buffer (pH 7.5), 0.5 ml 3 % Tx-100/0.075 % SDS in extraction buffer was added, and the lysate was centrifuged as described before. The pellet containing NPCs and nuclear lamina was suspended in 500 μl 0.3 % EmpigenBB (Sigma)/ in extraction buffer (pH 7.5) and incubated for 10 min on ice. The insoluble nuclear lamina was removed by 15 min centrifugation at 4°C and 15,000 x g.

#### Microinjection

1.5 x 10^5^ OR-EGFP-U2OS cells were seeded into 35mm plates and cytoplasmic microinjection was performed using an Eppendorf FemtoJet 4 and Injectman N12 coupled to a Zeiss Observer Z1 microscope. The medium was changed by CO_2_ independent medium (Gibco), and the injection solution contained 200 ng/μl of plasmid bearing the ANCH-mCherry sequence (approximately 160 copies/cell) in PBS. Prior to injection, the solution was centrifuged for 15 min, 13000 x g to avoid precipitates/aggregates.

#### Cell permeabilisation and chromatin digestion

U2OS-OR-EGFP cells (7.5 x 10^3^) were seeded onto Slide 8 well (Ibidi, 80826). One day after, the cells were transduced with 100 vg/cell scAAV2-ANCH-mCherry for 24 hrs. The chromatin of the cells was stained with 2 μg/mL Hoechst 33342 in pre-warmed DMEM for 30 min, before rinsing and submergence in 100 μL transport buffer (2 mM Mg-acetate, 20 mM HEPES, 110 mM K-acetate, 1 mM EGTA, 5 mM Na-acetate, 2 mM DTT, 1 mM protease-inhibitor cocktail, pH = 7.3) per well. Cells with visible scAAV2 genomes were then analysed by LSM (with CO_2_, H_2_O saturation, 37°C), followed by addition of digitonin at 80 μg/mL (Abcam ab141501) dissolved in transport buffer pre-warmed to 37°C. After ∼5-10 min of cell permeabilisation, digitonin was deactivated by the addition of 10 % FCS, followed by rinsing with transport buffer. Finally, 2.5 U/μL benzonase was added in 100 μL transport buffer. Images were taken in z-stacks of 5 optical slices over a total of 4 μm. Images were recorded every 30 s for 20 min. After time-lapse microscopy, cells were fixed as previously described and z-stacks of 32 optical sections totalling 9 μm were recorded. 3D reconstruction was performed using a macro in Image J.

#### Cellular fractionation

1 x 10^6^ cells were pre-treated with thapsigargin or DMSO for 1 h and transduced with 100 vg/cell of scAAV2-EYFP for 2 h in the presence of the inhibitor. The medium was then changed to DMEN with inhibitor. After 20 h the cells were collected and counted to obtain and adjust the cell number for subcellular fractionation. Cellular fractionation was done using a commercial kit (Thermo Fisher kit, ref. 78840), followed by digital droplet PCR. To test the purity of the fractions, Western blot was performed by mixing 25 μg of each fraction with Laemmli sample buffer/ β-mercaptoethanol. The samples and 5 μl of ECL rainbow maker full range (Amersham) were loaded onto mini-protean TGX stain free gel (Bio-Rad) using Tris/Glycine/SDS buffer (Bio-Rad). Proteins were transferred using Trans-Blot Turbo Transfer Pack, 0.2 μm PVDF, with Trans-Blot Turbo Transfer Buffer (Bio-Rad), using the Trans-Blot Turbo Transfer System (Bio-Rad). The membrane was then blocked for 1 h with 10% FCS in TBS/ 0.1% Tween 20 (TBS-T) and incubated with primary antibodies (1/2000 histone H3 (D1H2) XP® rabbit mAb #4499, 1/1000 nucleoporins MAb414 and 1/3000 lamin A/C conjugated with *horseradish peroxidase* (HRP) in TBS-T/1% FCS/1% NP40 overnight, at 4 C. The membrane was washed three times with 1X TBS-T, 10 min, then incubated with 1/40.000 rabbit anti-mouse HRP (DAKO) for 1 h, at RT, followed by three washes with TBS-T for 10 min and once with TBS. Proteins were incubated with ECL prime western blotting detection reagents (Amersham) for 5 min and visualised using ChemiDoc MP and imaging light conv. screen (Bio-Rad).

#### Degradation of scAAV2-EYFP genome by DNase I

1 x 10 ^7^ vg/sample of scAAV2-EYFP was mixed and incubated with 2 mM CaCl_2_, or 2.5 μg nucleoporins, or 2 mM CaCl_2_ + 2.5 μg nucleoporins, or 2 mM CaCl_2_ + 2.5 μg nucleoporins + 100 μg/ml WGA for 1 h at 37°C. As a control for capsid disintegration, the vector was heated at 75°C for 30 min. 6 μg of DNase I was added to the samples and incubated at 37°C for 1 h. After incubation, the samples were treated with 10 mM of EDTA and kept on ice. Genome extraction and quantification was performed using Magna Pure and droplet digital PCR.

### Quantification and statistical analysis

Statistical analyses were performed using GraphPad. The statistical test is described in each section of STAR Methods when quantification was applied. The value of n means the number of cells and is located on the top of the figures and the description of what n represents is given in figure legend as well as definition of centre and precision measures.
