# Supplementary material for: Real-time genome imaging of host interactions in adeno-associated virus genome release
Source: iScience. 2025 May 8;28(6):112624. doi: 10.1016/j.isci.2025.112624 (PMC12178803; doi:10.1016/j.isci.2025.112624)
Supplement: Document S1. Figures S1–S6 and Table S1 [file mmc1.pdf]

iScience, Volume *28*

Supplemental information

Real-time genome imaging

# of host interactions in adeno-associated

virus genome release

Luisa F. Bustamante-Jaramillo, Lei Yue, Joshua Fingal, Gustaf Rydell, Maria Johansson, Tomas Edreira, Oliver J. Müller, Susanne Hille, Martin Müller, Franck Gallardo, Qingxin Chen, Marie-Lise Blondot, and Michael Kann

A

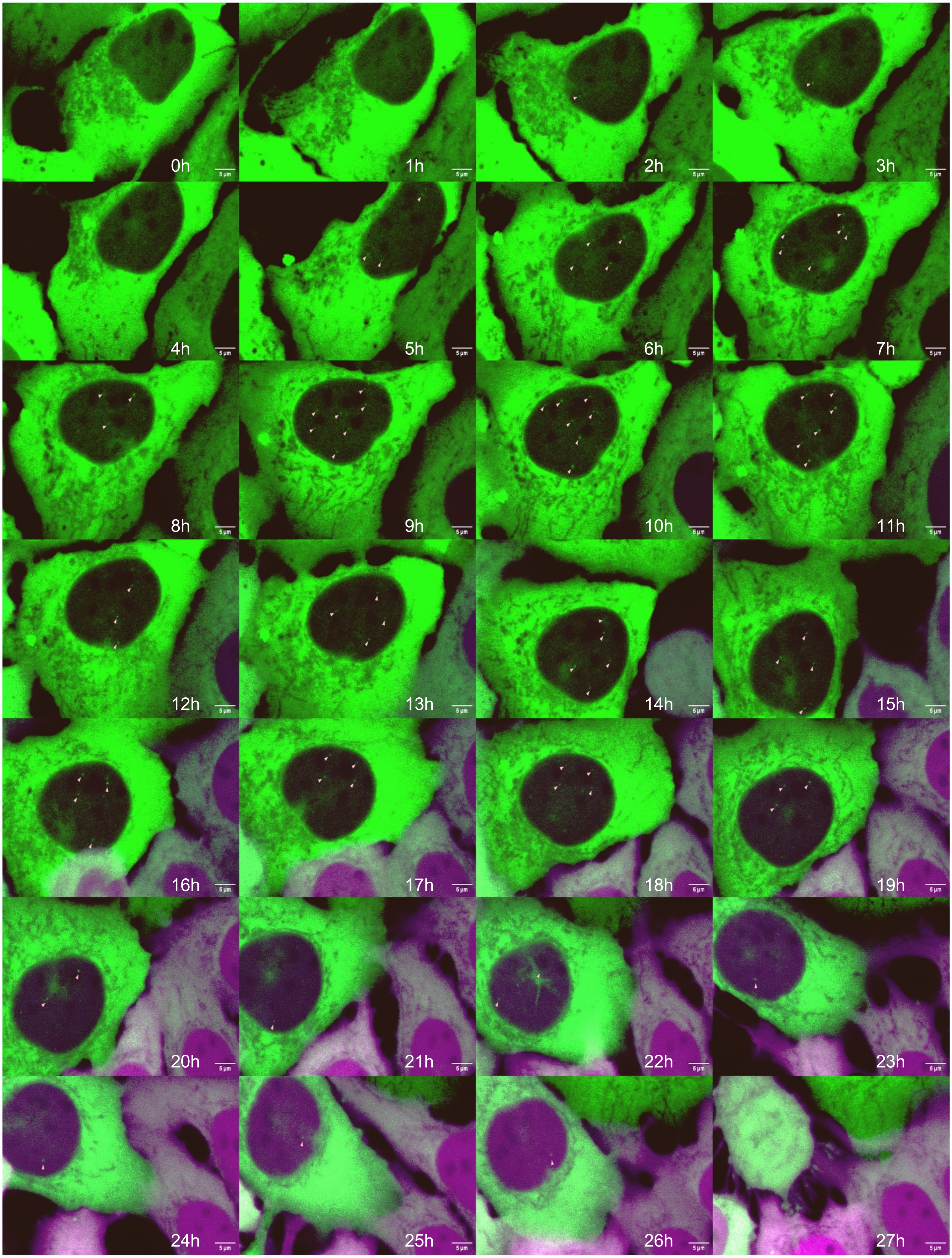

Figure S1. Visualisation of scAAV2-ANCH-mCherry genomes and mCherry expression in U2OS-OR-GFP cells. A) Time lapse images from Video 1. White arrows indicate the scAAV2 genomes and mCherry expression is shown in magenta.

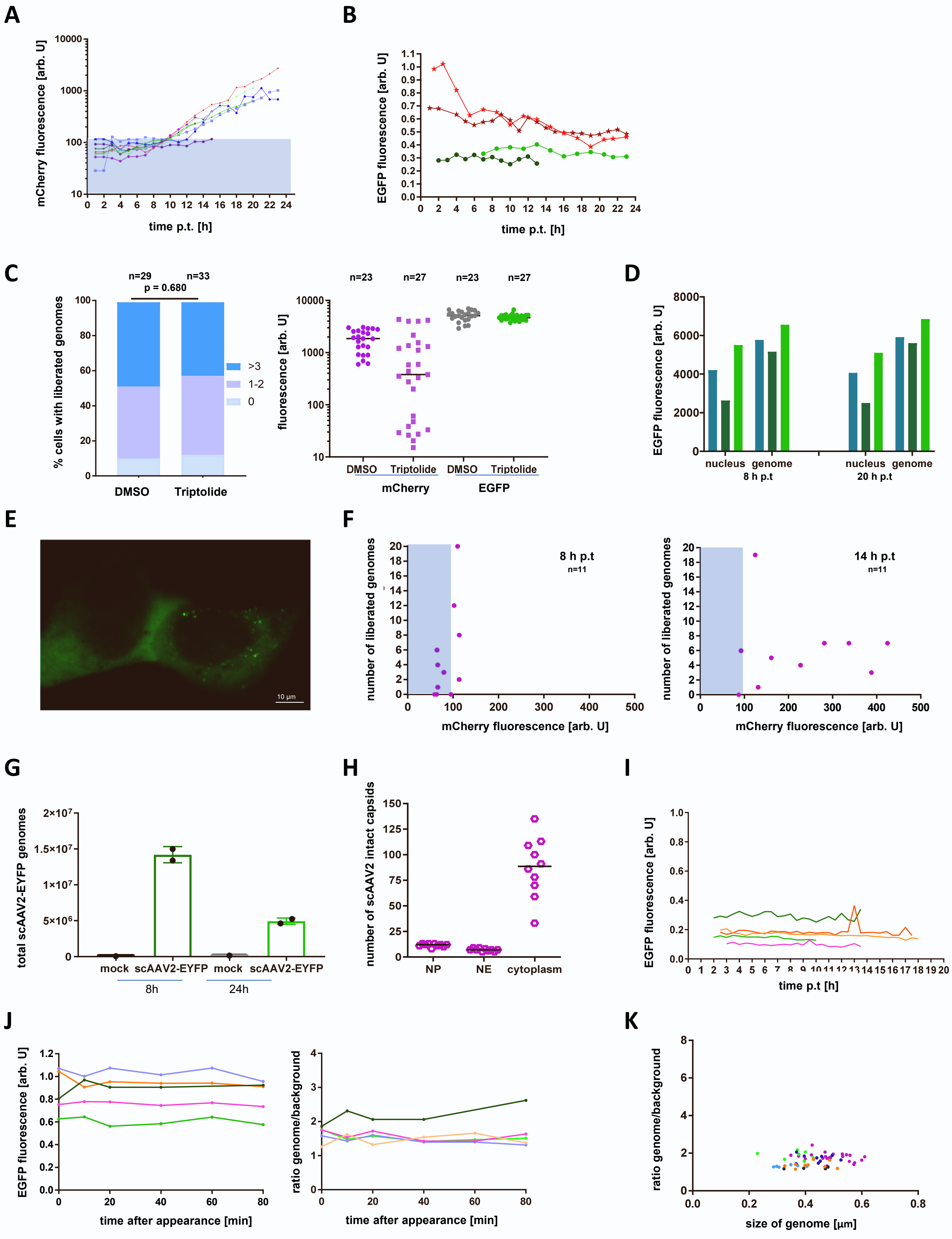
A
mCherry fluorescence [arb. U]
time p.t. [h]
B
EGFP fluorescence [arb. U]
time p.t. [h]
C
n=29
n=33
p = 0.680
% cells with liberated genomes
>3
1-2
0
DMSO
Triptolide
n=23
n=27
n=23
n=27
fluorescence [arb. U]
DMSO
Triptolide
DMSO
Triptolide
mCherry
EGFP
D
EGFP fluorescence [arb. U]
nucleus
genome
8 h p.t
nucleus
genome
20 h p.t
E
10 µm
F
number of liberated genomes
8 h p.t
n=11
mCherry fluorescence [arb. U]
number of liberated genomes
14 h p.t
n=11
mCherry fluorescence [arb. U]
G
total scAAV2-EYFP genomes
mock
scAAV2-EYFP
mock
scAAV2-EYFP
8h
24h
H
number of scAAV2 intact capsids
NP
NE
cytoplasm
I
EGFP fluorescence [arb. U]
time p.t [h]
J
EGFP fluorescence [arb. U]
time after appearance [min]
ratio genome/background
time after appearance [min]
K
ratio genome/background
size of genome [µm]

Figure S2. Visualisation of scAAV2-ANCH-mCherry genomes and mCherry expression in U2OS-OR-GFP cells. A) Quantification of mCherry expression over time (8 cells; 4 experiments). X-axis: time [h], y-axis: mCherry expression. Each dot/colour represents one cell. The blue shadow represents the background. **B)** Bleaching of two OR-EGFP aggregates (dark and light red) and bleaching resistance of genome-derived foci (dark and light green), from different time serie. X-axis: time after transduction [h]; y-axis: EGFP intensity. **C)** Left panel: quantification of released genomes at 16 h p.t. when triptolide 1 μM was added and after 5 h later upon triptolide at 1 μM. The p-value on top of the bars indicates the non-significant difference. Right panel: quantification of EGFP intensity of genomes and mCherry expression upon triptolide-treatment (quantification from 7 images, n = number of cells, the black bar is the median). **D)** Quantification of EGFP signals in the whole nuclei and quantification of one genome in the corresponding nucleus (3 cells; blue, dark green, green bars) at 8 and 20 h p.t. **E)** Detection of plasmids comprising the scAAV2-ANCH-mCherry genome microinjected into the cells (right cell; epifluorescence microscopy. Scale bar 10 μm). **F)** Quantification of mCherry fluorescence (x-axis) and number of released genomes (y-axis) in the same cells at 8 h and 14 h p.t. The blue shadow represents the background. **G)** Quantification of scAAV2-EYFP genomes in isolated nuclei at 8 h and 24 h p.t. by droplet digital PCR. The error-bars indicate standard-deviation from duplicates. **H)** Quantification of number of intact capsids (A20 antibody) in nucleus (NP), Nucler envelope (NE) and cytoplasm at 2h p.t. (10 cells). The black bar is the median. **I)** Intensity of genome fluorescence over time, (example of 5 foci in 5 cells from 3 experiments). X-axis: time [h]; y- axis: intensity. Each line represents one genome. **J)** Intensity of released genome fluorescence after first appearance. Left: crude intensity, right: normalised by intranuclear OR_EGFP fluorescence. **K)** Fluctuation of genome size during time-lapse. X-axis: size of 6 random genomes overtime from 5 cells, from 3 experiments ; Y-axis: genome intensity normalised by intranuclear OR_EGFP fluorescence.

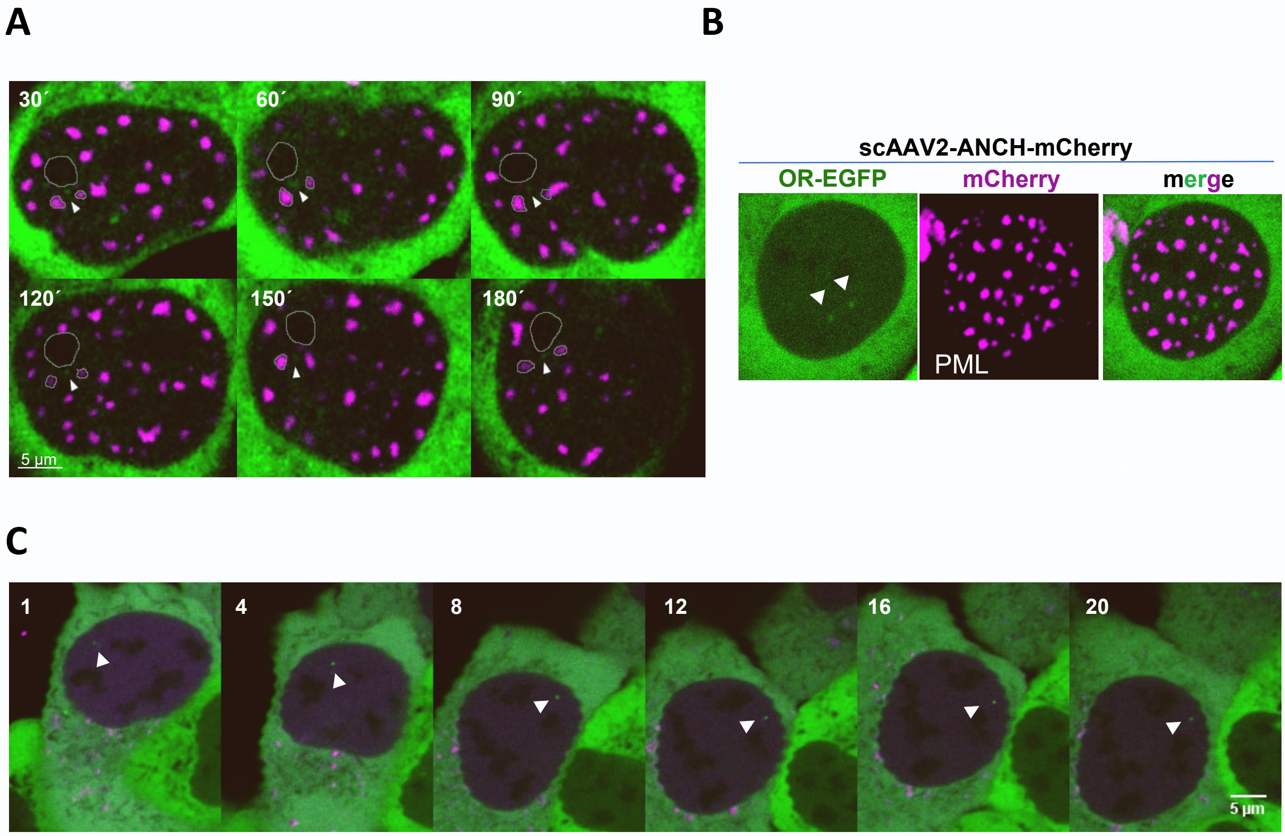

Figure S3. Immobility of scAAV2-ANCH-mCherry plasmids and released scAAV2-ANCH-mCherry genomes. **A)** U20S-OR-EGFP cells expressing PML in magenta and transduced with scAAV2-ANCH-mCherry. Time course, 6 frames, 30 min per frame. The time-lapse starts at 2 h p.t. The nucleolus and two PML foci are indicated to monitor the movement of the cells. The white arrow shows the position of the scAAV2 genome. **B)** Localisation of released genomes after scAAV2-ANCH-mCherry-transduction relative to marker proteins found in stationary subcellular structures. (PML-mCherry). **C)** Foci 20 h after transfection of scAAV2-ANCH-mCherry plasmids. LSM images taken at the indicated time (h). White arrow indicate the plasmid. Scale bar 5 µm.

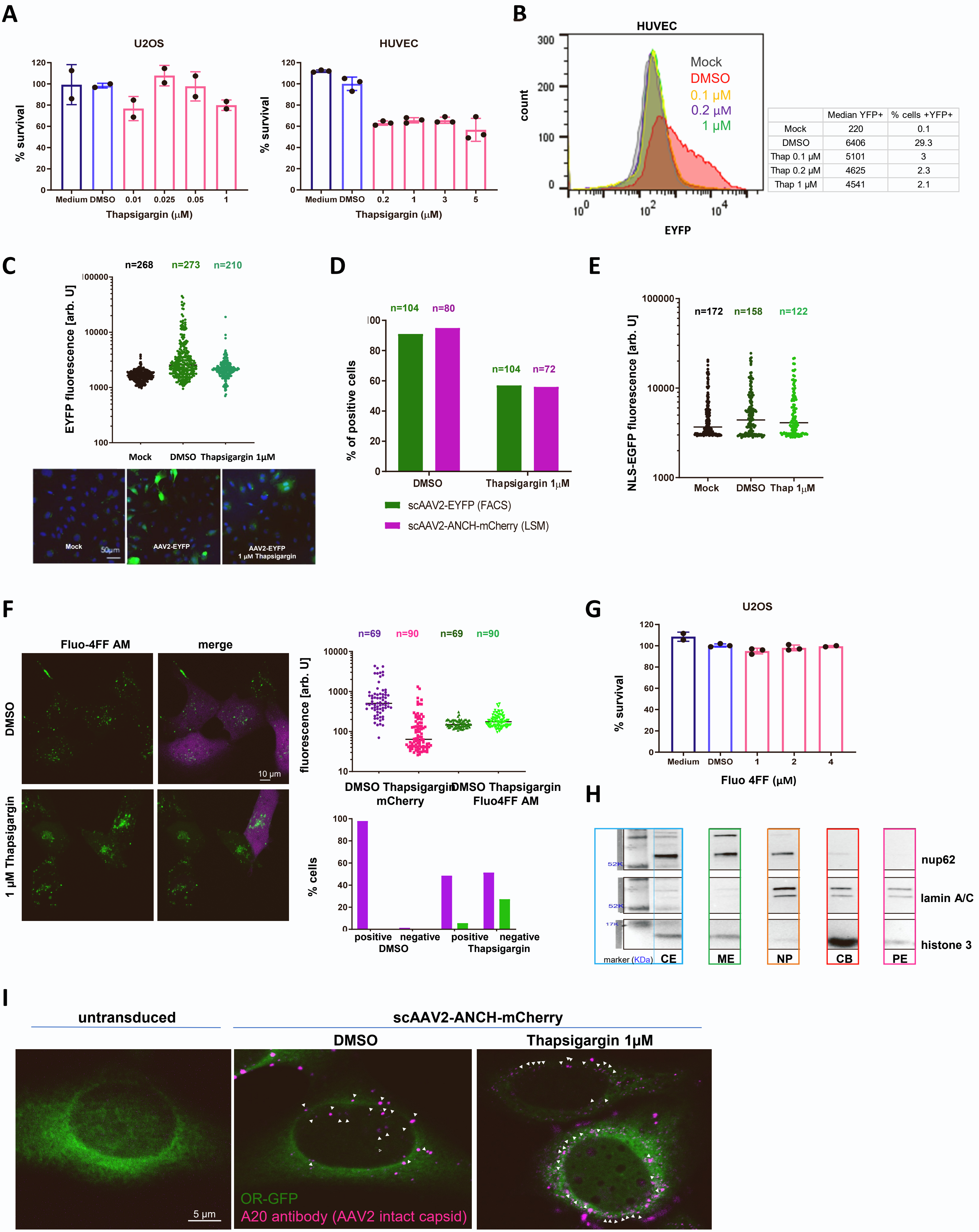

	Median YFP+	% cells +YFP+
Mock	220	0.1
DMSO	6406	29.3
Thap 0.1 µM	5101	3
Thap 0.2 µM	4625	2.3
Thap 1 µM	4541	2.1

Figure S4. Effect of Ca^{++} in genome release. **A)** Cell viability at different Thapsigargin concentrations (red). X-axis: Thapsigargin concentration. Left panel: U2OS cells, right panel: HUVEC cells. Dark blue: medium, light blue: DMSO (Thapsigargin solvent). The bars indicate the standard deviation from replicates. **B)** Transduction of HUVEC cells by scAAV2-EYFP in the presence of 0.1 μM (orange), 0.2 μM (magenta) and 1 μM (green) Thapsigargin or in the presence of DMSO (red) by FACS analysis. Mock are non-transduced cells (upper panel) 24 h p.t. Lower panel: quantification. **C)** as in **B)** but analysed by LSM. the black bar is the median. Lower panel: LSM image; upper panel: mCherry quantification, n = number of cells. Scale bar 50 μm. **D)** scAAV2-EYFP-transduction for 1h (green, FACS analysis) and scAAV2-ANCH-mCherry-transduction (magenta, quantification of LSM images) of U2OS cells in the presence of Thapsigargin (right) and DMSO (left) at 24 h p.t. n: number of cells analysed by LSM. **E)** Quantification of NLS-EGFP-expression stalely expressed in HuH-7 cells in the presence of 1 μM Thapsigargin or DMSO for 24 h. Quantification after LSM. The black bar is the median **F)** Ca^{++} distribution in scAAV2-mCherry-transduced U2OS cells at 1 μM Thapsigargin or DMSO 24 h p.t. Left panels: LSM images of Fluo4-FF, AM (green) and mCherry (red). Scale bar 10 μm. Right upper panel: individual values showing the intensity of mCherry and OR-EGFP upon 1 μM Thapsigargin and DMSO treatment. The black bar is the median. Right lower panel: magenta: percentage of mCherry-positive and -negative cells. Green: percentage of cells with diffuse Fluo-4FF, AM signal. Cells were either Thapsigargin- or DMSO-treated for 24 h. **G)** viability assay of U2OS upon addition of Fluo-4FF, AM (red). Dark blue: medium, light blue: DMSO (Fluo-4FF, AM solvent). The bars indicate the standard deviation from replicates. **H)** Control of cellular fractionation. Immune blots using the antibodies indicated on the right. Molecular weight size marker (KDa) indicated on the left. CE: cytosolic fraction, ME: membrane fraction, NP: nucleoplasmic fraction, CB: chromatin binding protein, PE: cytoskeleton fraction. **I)** Inmunostain with A20 antibody in DMSO and Thapsigargin treated cells after 2h p.t. with scAAV2-ANCH-mCherry. White arrows indicate the AAV2 capsids. Scale bar 5 μm.

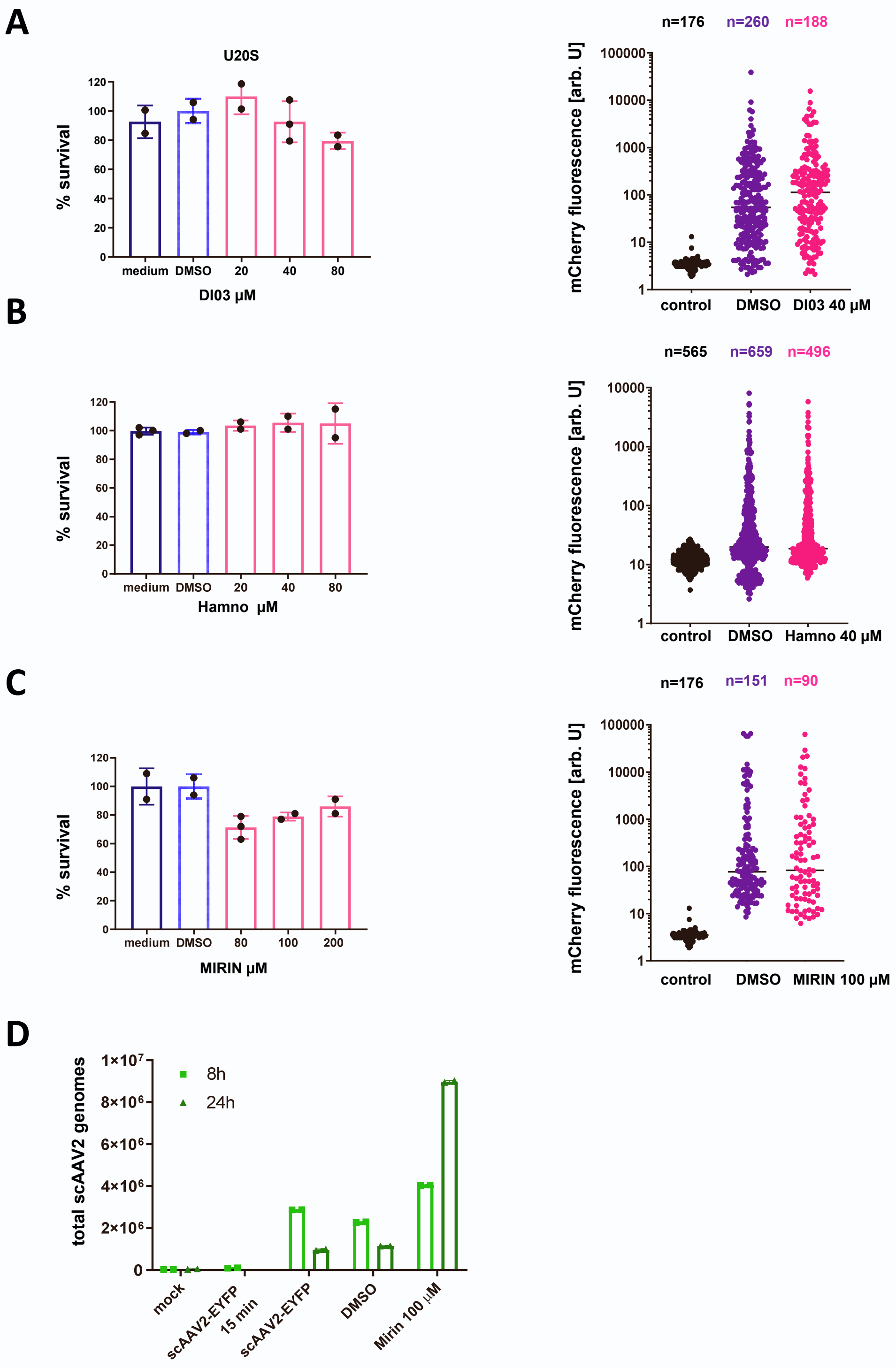

Figure S5. A) Left panel: viability assay of U2OS cells treated with the Rad52 inhibitor D-I03 at 20, 40 and 80 µM (red bars). Dark blue: medium, light blue: DMSO (D-I03 solvent). Right panel: Quantification of LSM images of mCherry expression in scAAV2-ANCH-mCherry plasmid-transfected cells upon D-I03 treatment (individual values, the black bar is the median). Control: untransfected cells. Y-axis: mCherry fluorescence in arb. U. The number of cells is given on top. **B)** Viability assay of cells treated with Hamno at 20, 40 and 80 µM. Right panel: is in A. **C)** Left panel: Viability assay of cells treated with MIRIN at 80, 100 and 200 µM (red bars). Dark blue: medium, light blue: DMSO (MIRIN solvent). Right panel: is in A), right panel. **D)** Quantification of scAAV2-EYFP genomes from isolated nuclei by droplet PCR 8 h and 24 h p.t. Cells were pre-treated with 100 µM MIRIN for 1 h and transduced with scAAV2-EYFP for 1 h. scAAV2-EYFP 15 min: nuclei extraction at 15 min p.t., prior to nuclear scAAV2-EYFP genome arrival. The error-bars indicates standard-deviation from replicates.

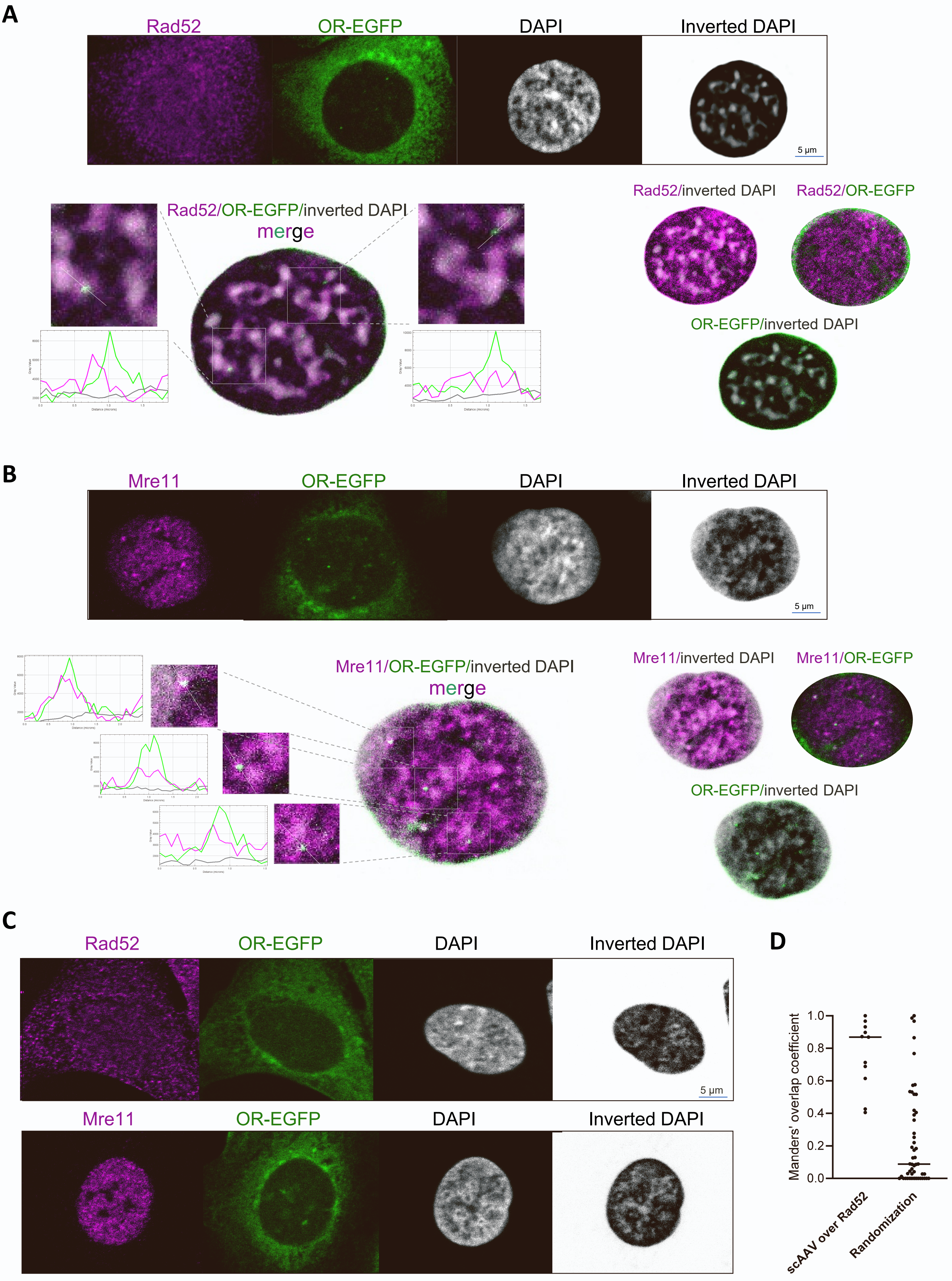
A
Rad52
OR-EGFP
DAPI
Inverted DAPI
5 µm
Rad52/OR-EGFP/inverted DAPI
merge
Rad52/inverted DAPI
Rad52/OR-EGFP
OR-EGFP/inverted DAPI
B
Mre11
OR-EGFP
DAPI
Inverted DAPI
5 µm
Mre11/OR-EGFP/inverted DAPI
merge
Mre11/inverted DAPI
Mre11/OR-EGFP
OR-EGFP/inverted DAPI
C
Rad52
OR-EGFP
DAPI
Inverted DAPI
5 µm
Mre11
OR-EGFP
DAPI
Inverted DAPI
D
Manders' overlap coefficient
1.0
0.8
0.6
0.4
0.2
0.0
scAAV over Rad52
Randomization

Figure S6. A) Localisation of Rad52 (in magenta) in scAAV2-ANCH-mCherry transduced and fixed cells at 24 h. scAAV2 genomes are in green. The top panel shows the individual channels and inverted DAPI, which was used for merging. The squares show the amplified area used for quantification where the white line was drawn to apply the plot profile. **B)** Same as A, but localisation of Mre11. **C)** Localisation of Rad52 and Mre11 in untransduced and fixed cells at 24 h. Scale bar 5 µm. **D)** Colocalization analysis of scAAV2 genomes with Rad52. Each dot represents the Manders' overlap coefficient calculated for a single scAAV2 genome over Rad52 signal in the cell. These values are compared against a distribution expected by chance in a randomization analysis.

mCherry intensity						
	cell1		cell2		cell3	
parent cell	68		540		562	
	1h p.d.	6h p.d.	1h p.d.	6h p.d.	1h p.d.	6h p.d.
daughter cell 1	73	93	917	1521	700	832
daughter cell 2	70	92	942	1443	692	812

Table S1. mCherry expression in parent and daughter cells. Quantification of mCherry fluorescence in the parent cells 1 h before division, in daughter cells 1 h and 6 h post division.
